# Inactivation *Rap2a* in Endothelial Cell Prevents Pulmonary Fibrosis by Regulating Immune Microenvironment Through MAP4K4‐VCAM1 Signaling

**DOI:** 10.1002/advs.202519892

**Published:** 2026-02-11

**Authors:** Xiaolan Zheng, Peng Yue, Kaiyu Zhou, Guidong Gong, Yue Zhang, Sha Lin, Xu Liu, Yanjiang Zheng, Siyuan Jing, Junling Guo, Yan Qi, Bi‐Sen Ding, Yimin Hua, Yifei Li

**Affiliations:** ^1^ Key Laboratory of Birth Defects and Related Diseases of Women and Children of MOE Department of Pediatrics West China Second University Hospital Sichuan University Chengdu Sichuan China; ^2^ Institute of Cardiovascular Surgery The Second Affiliated Hospital Army Medical University Chongqing China; ^3^ BMI Center for Biomass Materials and Nanointerfaces College of Biomass Science and Engineering Sichuan University Chengdu Sichuan China; ^4^ State Key Laboratory of Biocatalysis and Enzyme Engineering School of Life Science Hubei University Wuhan Hubei China; ^5^ Key Lab of Birth Defects and Related Diseases of Women and Children of MOE State Key Lab of Biotherapy State Key Laboratory of Respiratory Health and Multimorbidity West China School of Basic Medical Sciences & Forensic Medicine West China Second University Hospital Sichuan University Chengdu Sichuan China; ^6^ Department of Pediatric Cardiovascular The Second Hospital & Clinical Medical School Lanzhou University Lanzhou Gansu China

**Keywords:** CellNex, endothelial cells, immune microenvironment, pulmonary fibrosis, Rap2a

## Abstract

Idiopathic pulmonary fibrosis (IPF) is characterized by progressive fibrotic remodeling accompanied by persistent endothelial activation and leukocyte infiltration. Although endothelial dysfunction is increasingly recognized as a key contributor to fibrogenesis, the intracellular signaling pathways that couple inflammatory cues to endothelial–immune interactions remain incompletely defined. Ras‐related protein Rap2a (RAP2A), a small GTPase implicated in stress and inflammatory signaling, has not been systematically investigated in pulmonary endothelial cells during fibrotic lung injury. Here, using a bleomycin‐induced experimental lung fibrosis model, we observed that RAP2A expression was markedly upregulated in pulmonary endothelial cells and correlated with disease severity. Endothelium‐enriched knockdown of Rap2a via AAV9‐Cdh5‐shRNA attenuated inflammatory cell adhesion to the pulmonary endothelium, reduced fibrotic remodeling, and improved lung function. Mechanistically, RAP2A promoted endothelial activation by enhancing MAP4K4‐dependent signaling and upregulating vascular cell adhesion molecule 1 (VCAM1) in response to pro‐inflammatory stimulation, thereby facilitating leukocyte–endothelial interactions. In vitro assays further demonstrated that RAP2A deficiency impaired tumor necrosis factor‐α–induced endothelial adhesiveness without affecting basal endothelial integrity. Collectively, our findings identify endothelial RAP2A as a regulator of inflammatory endothelial activation in experimental lung fibrosis and suggest that targeting RAP2A‐mediated signaling may represent a potential strategy to modulate endothelial–immune crosstalk during fibrotic lung injury.

## Graphical abstract

We identified the endothelial RAP2A as a regulator of inflammatory endothelial activation in experimental lung fibrosis and suggest that targeting RAP2A‐mediated signaling may represent a potential strategy to modulate endothelial–immune crosstalk during fibrotic lung injury. Thus, we induced Erythrocyte_nex_‐based nanocarriers targeting suppressing endothelial *Rap2a*, which alleviated pulmonary fibrosis in vivo.

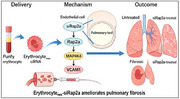

## 1 Introduction

Idiopathic pulmonary fibrosis (IPF) is a devastating interstitial lung disease characterized by progressive extracellular matrix (ECM) accumulation and ultimately fatal respiratory failure [1, 2]. Although fibroblast activation and alveolar epithelial injury have long been regarded as central drivers of IPF pathogenesis, accumulating evidence indicates that vascular endothelial cells (ECs) actively participate in disease progression rather than functioning as passive bystanders [2, 3]. In both human IPF specimens and experimental animal models, ECs exhibit features of immune activation, increased permeability, and dysregulated angiocrine signaling [1, 2]. Notably, the pulmonary vascular niche plays a critical role in orchestrating immune cell recruitment and shaping a pro‐fibrotic immune microenvironment.

Despite these advances, the upstream endothelial regulators governing maladaptive immune–vascular interactions remain incompletely understood, particularly with respect to intercellular communication among endothelial cells, immune cells, and fibroblasts [3, 4]. Previous studies have implicated canonical pathways, including TGF‐β/Smad, VEGF, and Notch signaling, in pathological vascular remodeling; however, comparatively little attention has been paid to how endothelial cells sense and respond to ECM remodeling and increased tissue stiffness during fibrotic progression. Small GTPases function as molecular switches that integrate biochemical and mechanical cues to regulate cellular responses, yet their role in coordinating endothelial activation during fibrotic lung injury remains largely unexplored [5, 6].

RAP2A (encoded by *Rap2a*), a member of the Ras‐related small GTPase family, has been implicated in cytoskeletal organization and cell–cell junction regulation. Previous studies have identified RAP2A as a modulator of Hippo pathway signaling and have linked it to diverse immune‐regulatory processes [7, 8, 9]. Notably, Noriko et al. [7], demonstrated that RAP2A activates JNK signaling via MAP4K4 in non‐pulmonary cellular contexts under inflammatory conditions. However, whether endothelial RAP2A contributes to fibrotic lung pathology by modulating inflammatory endothelial activation and immune–vascular crosstalk has not been systematically investigated.

In this study, we examined the role of endothelial RAP2A in experimental pulmonary fibrosis, focusing on its function in coupling pro‐inflammatory stimulation to endothelial activation and leukocyte adhesion. Using complementary approaches, including endothelial‐targeted *Rap2a* genetic deletion and cell‐type‐enriched AAV9‐mediated gene manipulation in bleomycin‐induced lung fibrosis models, we demonstrate that RAP2A critically regulates inflammatory endothelial responses and fibrotic progression. Single‐cell transcriptomic profiling combined with CellChat‐based communication analysis further identifies RAP2A as a modulator of endothelial–immune signaling, particularly in pathways associated with macrophage‐derived ligands. Mechanistically, RAP2A promotes endothelial activation by upregulating VCAM1 expression and enhancing inflammatory cell–endothelial interactions.

Furthermore, to explore the therapeutic relevance of targeting endothelial RAP2A, we employed a red blood cell–based siRNA delivery platform to achieve partial endothelial *Rap2a* silencing in vivo. This strategy resulted in attenuated pulmonary fibrosis and preservation of lung function in experimental models. Together, our findings establish endothelial RAP2A as a regulator of inflammatory endothelial activation in fibrotic lung injury and highlight its potential as a modulatory target for controlling immune–vascular interactions during pulmonary fibrosis.

## 2 Results

### 2.1 *RAP2A* Expression Was Elevated in IPF Lungs and Positive Associated With Pro‐Fibrotic Gene Signatures

**FIGURE 1:**
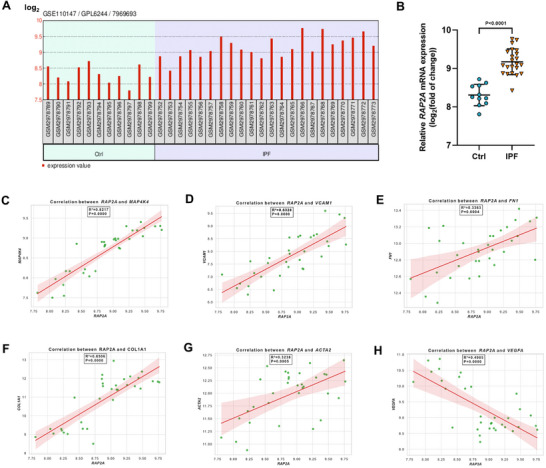
*RAP2A* expression is increased in IPF lungs and associated with pro‐fibrotic gene signatures. (A) *RAP2A* mRNA expression in control and IPF lung tissues from the GSE110147 dataset (log_2_‐normalized expression). Each dot represents one sample. (B) Quantification of *RAP2A* expression in control versus IPF lungs. Data are shown as mean ± SD. Statistical significance was assessed using a two‐tailed unpaired *t*‐test. Sample sizes: control (n = 11), IPF (n = 22). (C–H) Associations between *RAP2A* expression and indicated genes in IPF lung samples: *MAP4K4* (C), *VCAM1* (D), *FN1* (E), *COL1A1* (F), *ACTA2* (G), and *VEGFA* (H). Lines indicate best‐fit linear regression; shaded areas denote 95% confidence intervals. Correlation strength is reported as [Pearson r/Spearman ρ] with corresponding two‐tailed *P* values; R^2^ values are shown on the plots.

To evaluate the clinical relevance of *RAP2A* in pulmonary fibrosis, we first analyzed its expression in human lung transcriptomic datasets. Analysis of the GSE110147 dataset revealed that *RAP2A* mRNA levels were significantly increased in lung tissues from patients with IPF compared with non‐fibrotic controls (Figure 1A,B).

We next examined the transcriptional relationship between *RAP2A* and genes associated with fibrotic remodeling and endothelial activation. In IPF samples, *RAP2A* expression showed positive correlations with *MAP4K4* and *VCAM1*, genes involved in inflammatory signaling and leukocyte–endothelial interactions, as well as with classical fibrosis‐associated markers including *FN1*, *COL1A1*, and *ACTA2* (Figure 1C–G). In contrast, *RAP2A* expression was negatively correlated with *VEGFA* (Figure 1H), a key regulator of vascular homeostasis and angiogenic maintenance.

Together, these analyses demonstrate that *RAP2A* is upregulated in human IPF lungs and transcriptionally associated with pro‐fibrotic and endothelial activation–related gene signatures, supporting a potential link between *RAP2A* expression and pathological fibrotic remodeling.

### 2.2 *RAP2A* was Enriched in Endothelial Compartments in Animal Pulmonary Fibrosis Model

**FIGURE 2:**
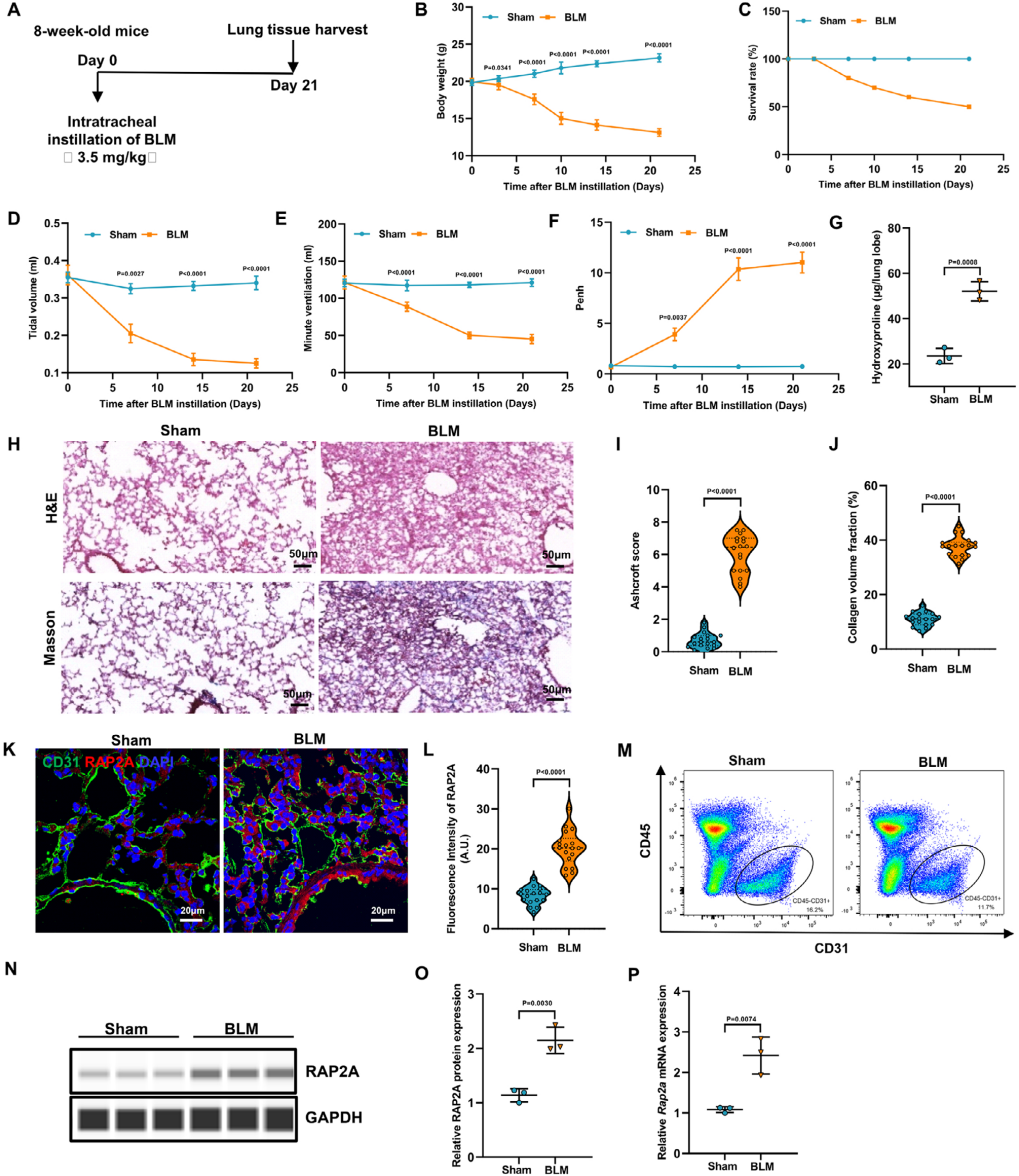
*RAP2A* is increased in pulmonary endothelial cells in bleomycin‐induced pulmonary fibrosis. (A) Experimental schematic of the bleomycin (BLM)‐induced pulmonary fibrosis model and tissue collection time points. (B) Body weight change and (C) Kaplan–Meier survival curves for sham and BLM‐treated mice (n = 3 per group). Body weight data are shown as mean ± SEM and analyzed by two‐way repeated‐measures ANOVA with Sidak's multiple‐comparisons test; survival was analyzed using the log‐rank (Mantel–Cox) test. (D–F) Lung function parameters, including tidal volume (TV), minute ventilation (MV), and enhanced pause (Penh) measured over 21 days post‐instillation (n = 6 per group). Data are mean ± SEM and were analyzed by two‐way repeated‐measures ANOVA with multiple‐comparisons correction. (G) Hydroxyproline content in lung tissue at day 21 (n = 3 per group), presented as mean ± SEM and analyzed using a two‐tailed unpaired *t*‐test. (H) Representative H&E and Masson's trichrome staining of lung sections at day 21 (scale bars, 50 µm). (I) Ashcroft score and (J) collagen volume fraction at day 21 (n = 3 per group; 6 fields per mouse). Ashcroft scores are shown as median with interquartile range and analyzed using the Mann–Whitney test; collagen volume fraction is shown as mean ± SEM and analyzed using a two‐tailed unpaired *t*‐test. (K,L) Immunofluorescence staining showing CD31 (green) and RAP2A (red) in lung sections at day 21 and quantification of RAP2A fluorescence intensity within CD31^+^ endothelial structures (n = 3 mice per group; 6 fields per mouse). Data are mean ± SEM and analyzed using a two‐tailed unpaired *t*‐test. (M) Flow cytometric gating strategy identifying pulmonary CD31^+^CD45^−^ endothelial cells from lung single‐cell suspensions at day 21. (N–P) RAP2A expression in flow‐sorted CD31^+^CD45^−^ endothelial cells collected at day 21, assessed by Western blot with densitometric quantification (N,O) and by qPCR (P) (n = 3 per group). Data are mean ± SEM and analyzed using a two‐tailed unpaired *t*‐test. Exact *P* values are indicated.

Intratracheal bleomycin (BLM) administration induced progressive clinical deterioration over the 21‐day observation period, as evidenced by body weight loss and reduced survival (Figure 2A–C). Pulmonary function testing revealed impaired lung mechanics, including decreased tidal volume and minute ventilation, accompanied by increased enhanced pause (Penh) values (Figure 2D–F). Consistent with fibrotic remodeling, biochemical and histopathological assessments demonstrated increased lung hydroxyproline content and marked architectural distortion on H&E and Masson's trichrome staining, leading to elevated Ashcroft scores and increased collagen volume fraction (Figure 2G–J).

We next examined the cellular distribution of RAP2A within the pulmonary vascular compartment. Immunofluorescence staining showed increased RAP2A signal within CD31^+^ endothelial structures in BLM‐treated lungs compared with vehicle controls, and quantitative analysis confirmed elevated endothelial RAP2A fluorescence intensity (Figure 2K,L). To further assess RAP2A expression specifically in endothelial cells, we identified and flow‐sorted the CD31^+^CD45^−^ endothelial population from lung single‐cell suspensions (Figure 2M). Immunoblotting and qPCR performed on the sorted CD31^+^CD45^−^ endothelial fraction consistently showed increased RAP2A protein and *Rap2a* mRNA levels after BLM challenge (Figure 2N–P), supporting endothelial enrichment of RAP2A upregulation during fibrotic lung injury.

In addition, Supplementary Figure S1A shows that the proportion of pulmonary CD31^+^CD45^−^ endothelial cells declined from week 1 to week 3 after BLM administration, consistent with endothelial loss or phenotypic alteration during fibrotic remodeling. Together with the day‐21 endothelial enrichment of RAP2A (Figure 2M–P) and the established fibrotic phenotype over the 21‐day course (Figure 2B–J), these data indicate that RAP2A is induced in pulmonary endothelial cells in experimental fibrosis and is observed in the setting of progressive structural and functional deterioration.

### 2.3 Endothelial‐Specific Inactivation of *Rap2a* Attenuated BLM‐Induced Pulmonary Fibrosis

**FIGURE 3:**
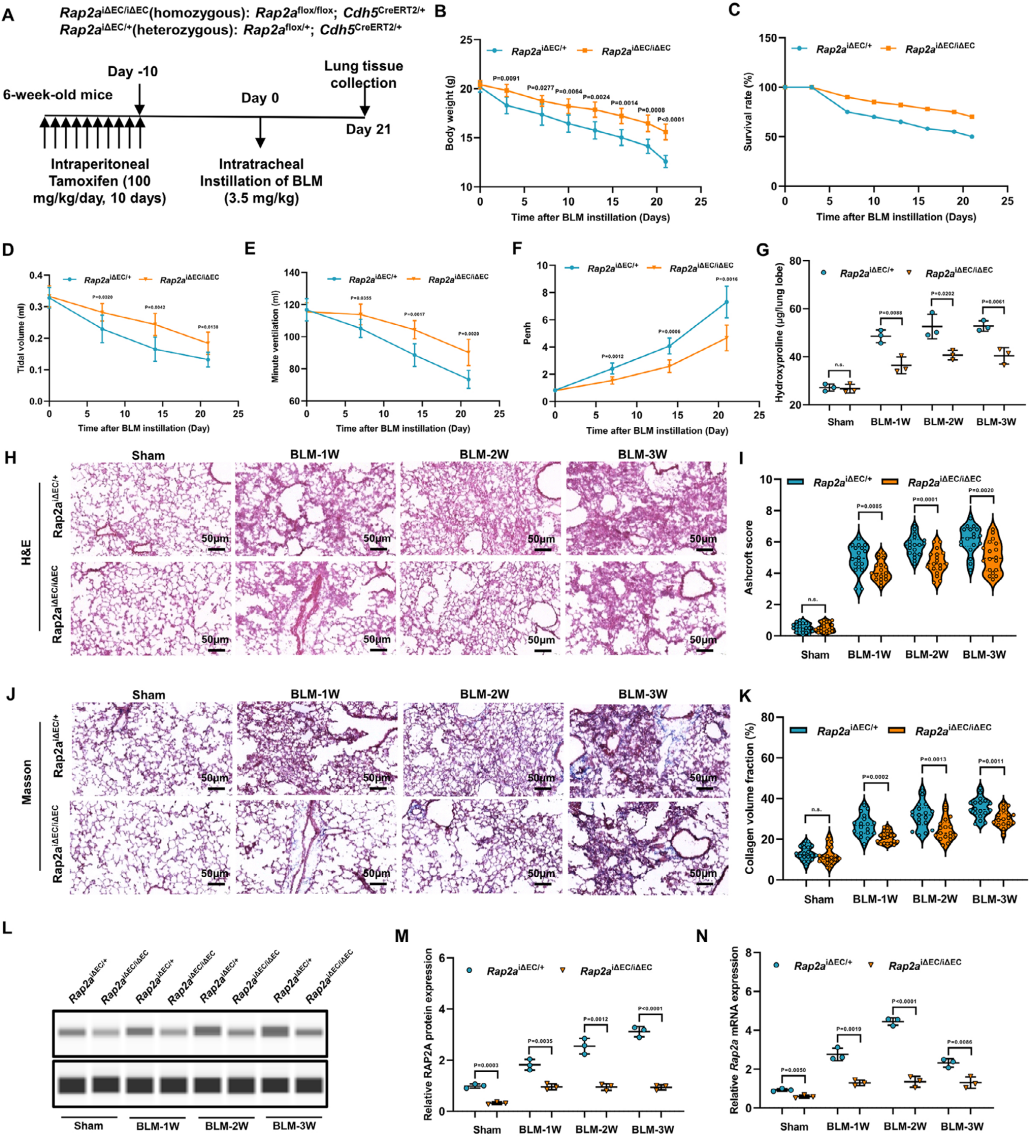
Endothelial‐specific inactivation of *Rap2a* mitigated BLM‐induced pulmonary fibrosis. (A) Schematic of tamoxifen‐induced endothelial Rap2a deletion and bleomycin (BLM) administration protocol. (B) Body weight change and (C) Kaplan–Meier survival curves in *Rap2a* ^ΔEC/+^ and *Rap2a* ^ΔEC/ΔEC^ mice following BLM challenge (n = 6 per group). Body weight data are presented as mean ± SEM and analyzed using two‐way repeated‐measures ANOVA with Sidak's multiple‐comparisons test; survival was analyzed using the log‐rank (Mantel–Cox) test. (D–F) Pulmonary function parameters, including tidal volume (D), minute ventilation (E), and enhanced pause (Penh) (F), were measured at days 7, 14, and 21 after BLM instillation (n = 6 per group). Data are presented as mean ± SEM and analyzed using two‐way repeated‐measures ANOVA with multiple‐comparisons correction. (G) Hydroxyproline content in lung tissue at 1, 2, and 3 weeks after BLM administration (n = 3 per group). Data are presented as mean ± SEM and analyzed using a two‐tailed unpaired *t*‐test. (H–K) Representative H&E (H) and Masson's trichrome (J) staining of lung sections at indicated time points, with corresponding quantification of Ashcroft score (I) and collagen volume fraction (K) (n = 3 per group; 6 fields per mouse). Ashcroft scores are presented as median with interquartile range (IQR) and analyzed using the Mann–Whitney test; collagen volume fraction is presented as mean ± SEM and analyzed using a two‐tailed unpaired *t*‐test. (L–N) RAP2A expression assessed in flow‐sorted pulmonary CD31^+^CD45^−^ endothelial cells (gating strategy shown in Figure S1B): representative Western blot (L) with densitometric quantification (M), and *Rap2a* mRNA expression determined by qPCR (N) (n = 3 per group). Data are presented as mean ± SEM and analyzed using a two‐tailed unpaired *t*‐test. Exact *P* values are indicated.

To investigate the functional role of endothelial *Rap2a* in fibrotic progression, we generated the tamoxifen‐inducible endothelial cell–specific *Rap2a* knockout mice (abbreviated as *Rap2a* ^ΔEC/ΔEC^) using the *Cdh5*‐CreERT2 system. The heterozygous littermates, which were genotyped as *Cdh5*‐CreERT2; *Rap2a* ^flox/+^ mice, received the same tamoxifen injection and served as controls (abbreviated as *Rap2a* ^ΔEC/+^). Mice were pre‐treated with tamoxifen for 10 days prior to BLM instillation and sacrificed on day 21 post BLM administration (Figure 3A).

Compared to heterozygous *Rap2a* ^ΔEC/+^ mice, homozygous *Rap2a* ^ΔEC/ΔEC^ mice exhibited significantly attenuated weight loss (Figure 3B), enhanced survival rates (Figure 3C), and preserved lung function as evidenced by significantly higher tidal volume (TV) and minute ventilation (MV), accompanied by reduced enhanced pause values over the experimental time course (Figure 3D–F). Hydroxyproline quantification revealed markedly diminished collagen accumulation in *Rap2a* ^ΔEC/ΔEC^ lungs at weeks 1, 2, and 3 following BLM challenge (Figure 3G). Histopathological examination revealed markedly attenuated fibrotic alterations in *Rap2a* ^ΔEC/ΔEC^ mice throughout all temporal phases following BLM administration, as demonstrated by H&E (Figure 3H) and Masson staining (Figure 3J). Quantitative morphometric analysis confirmed significantly reduced Ashcroft scores (Figure 3I) and collagen volume fraction (Figure 3K) in *Rap2a* ^ΔEC/ΔEC^ lungs compared to controls.

Efficient endothelial Rap2a deletion was verified by immunoblotting and qPCR analyses performed on flow‐sorted pulmonary CD31^+^CD45^−^ endothelial cells collected at the indicated time points. RAP2A protein levels were markedly reduced in endothelial cells isolated from *Rap2a* ^ΔEC/ΔEC^ mice compared with controls (Figure 3L,M), accompanied by decreased *Rap2a* mRNA expression (Figure 3N), confirming effective endothelial‐specific inactivation.

Consistent with preserved endothelial integrity, flow cytometric analysis revealed that *Rap2a* ^ΔEC/ΔEC^ lungs maintained a higher proportion of CD31^+^CD45^−^ endothelial cells during the 1–3 week period following BLM administration, whereas control mice exhibited a progressive decline in this population (Figure S1B). In parallel, immunofluorescence analysis demonstrated reduced α‐SMA–positive myofibroblast accumulation in *Rap2a* ^ΔEC/ΔEC^ lungs at later stages of fibrosis (weeks 1–3), with quantitative analysis confirming diminished α‐SMA signal intensity compared with controls (Figure S2).

Together, these results established that endothelial‐specific loss of *Rap2a* mitigates BLM‐induced pulmonary fibrosis, which is associated with preservation of the endothelial compartment, reduced fibrotic remodeling, and improved pulmonary function.

### 2.4 Endothelial *Rap2a* Depletion Attenuated Inflammatory Cell Infiltration and Cytokine Production in Fibrotic Lungs

**FIGURE 4:**
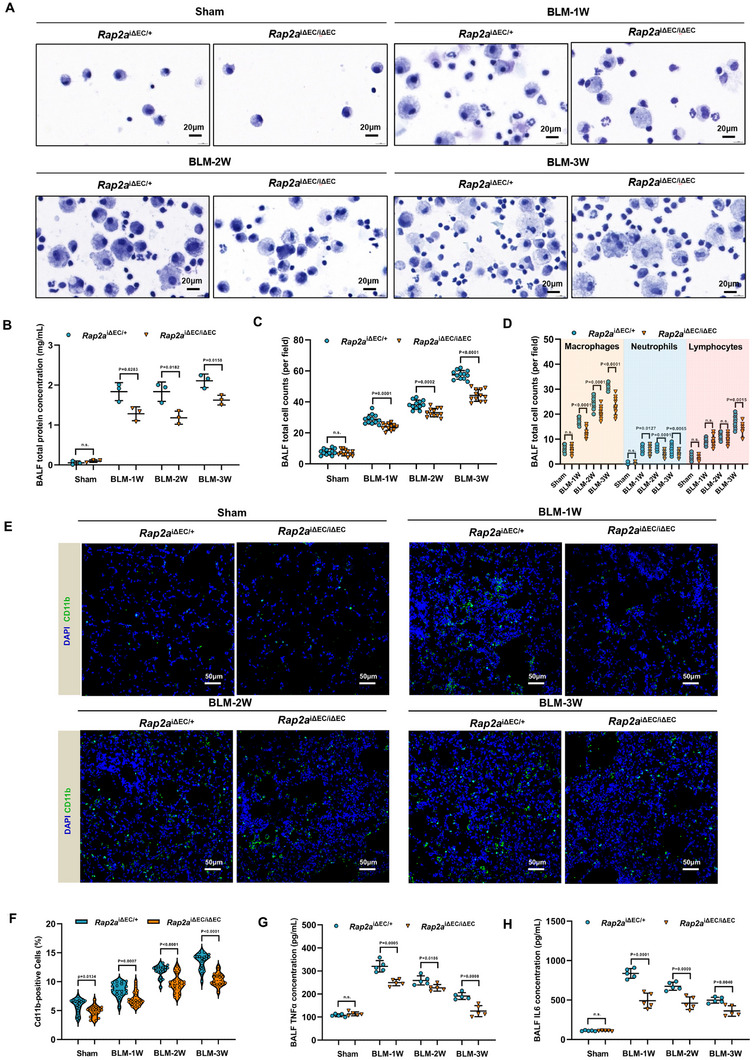
Endothelial‐specific depletion of *Rap2a* reduced immune cell infiltration and inflammation in the fibrotic lung. (A) Cytospin images of bronchoalveolar lavage fluid (BALF) cells from *Rap2a* ^ΔEC/+^ and *Rap2a* ^ΔEC/ΔEC^ mice at baseline and at 1, 2, and 3 weeks after bleomycin (BLM) administration. (B,C) Quantification of BALF total protein concentration (B) and total inflammatory cell counts (C) at baseline and at 1, 2, and 3 weeks post‐BLM (n = 3 per group). Data are presented as mean ± SEM and analyzed using two‐way repeated‐measures ANOVA with Sidak's multiple‐comparisons test. (D) Differential BALF cell counts showing macrophages, neutrophils, and lymphocytes at baseline and at 1, 2, and 3 weeks post‐BLM (n = 3 per group). Data are presented as mean ± SEM and analyzed using two‐way repeated‐measures ANOVA with multiple‐comparisons correction. (E) Representative immunofluorescence images of lung sections stained for CD11b (green) and DAPI (blue) at baseline and/or the indicated time points after BLM challenge. (F) Quantification of CD11b^+^ cell density in lung sections at the indicated time points (n = 3 per group; 6 fields per mouse). Data are presented as mean ± SEM and analyzed using two‐way repeated‐measures ANOVA with Sidak's multiple‐comparisons test. (G,H) BALF concentrations of TNF‐α (G) and IL‐6 (H) measured by ELISA at baseline and at the indicated time points after BLM administration (n = 5 per group). Data are presented as mean ± SEM and analyzed using two‐way repeated‐measures ANOVA with Sidak's multiple‐comparisons test. Exact *P* values are indicated.

To evaluate the contribution of endothelial Rap2a to inflammatory responses during bleomycin‐induced lung fibrosis, we analyzed bronchoalveolar lavage fluid (BALF) and lung tissues from *Rap2a* ^ΔEC/+^ and *Rap2a* ^ΔEC/ΔEC^ mice at defined time points following BLM challenge. Cytospin analysis revealed progressive accumulation of inflammatory cells in the BALF of *Rap2a* ^ΔEC/+^ control mice, whereas this response was attenuated in *Rap2a* ^ΔEC/ΔEC^ mice across the examined post‐injury period (Figure 4A). Quantitative measurements further demonstrated reduced BALF total protein levels and decreased total inflammatory cell counts in *Rap2a* ^ΔEC/ΔEC^ mice compared with controls (Figure 4B,C).

Differential leukocyte analysis showed that endothelial Rap2a deletion was associated with reduced numbers of macrophages, neutrophils, and lymphocytes in the BALF following BLM administration (Figure 4D). Consistently, immunofluorescence staining of lung sections demonstrated diminished CD11b^+^ myeloid cell accumulation in *Rap2a* ^ΔEC/ΔEC^ lungs, with quantitative analysis confirming a lower density of CD11b^+^ cells compared with *Rap2a* ^ΔEC/+^ mice (Figure 4E,F). In parallel, Figure S3 shows reduced accumulation of F4/80^+^ macrophages in Rap2a‐deficient lungs, particularly at weeks 2 and 3 after BLM challenge.

In addition to reduced inflammatory cell recruitment, analysis of BALF cytokine levels revealed significantly lower concentrations of TNF‐α and IL‐6 in *Rap2a* ^ΔEC/ΔEC^ mice compared with heterozygous controls (Figure 4G,H). Together, these data indicate that endothelial‐specific deletion of Rap2a is associated with attenuated leukocyte infiltration and reduced pro‐inflammatory cytokine production during experimental pulmonary fibrosis.

### 2.5 Endothelial *Rap2a* Overexpression Exacerbated BLM‐Induced Pulmonary Fibrosis

**FIGURE 5:**
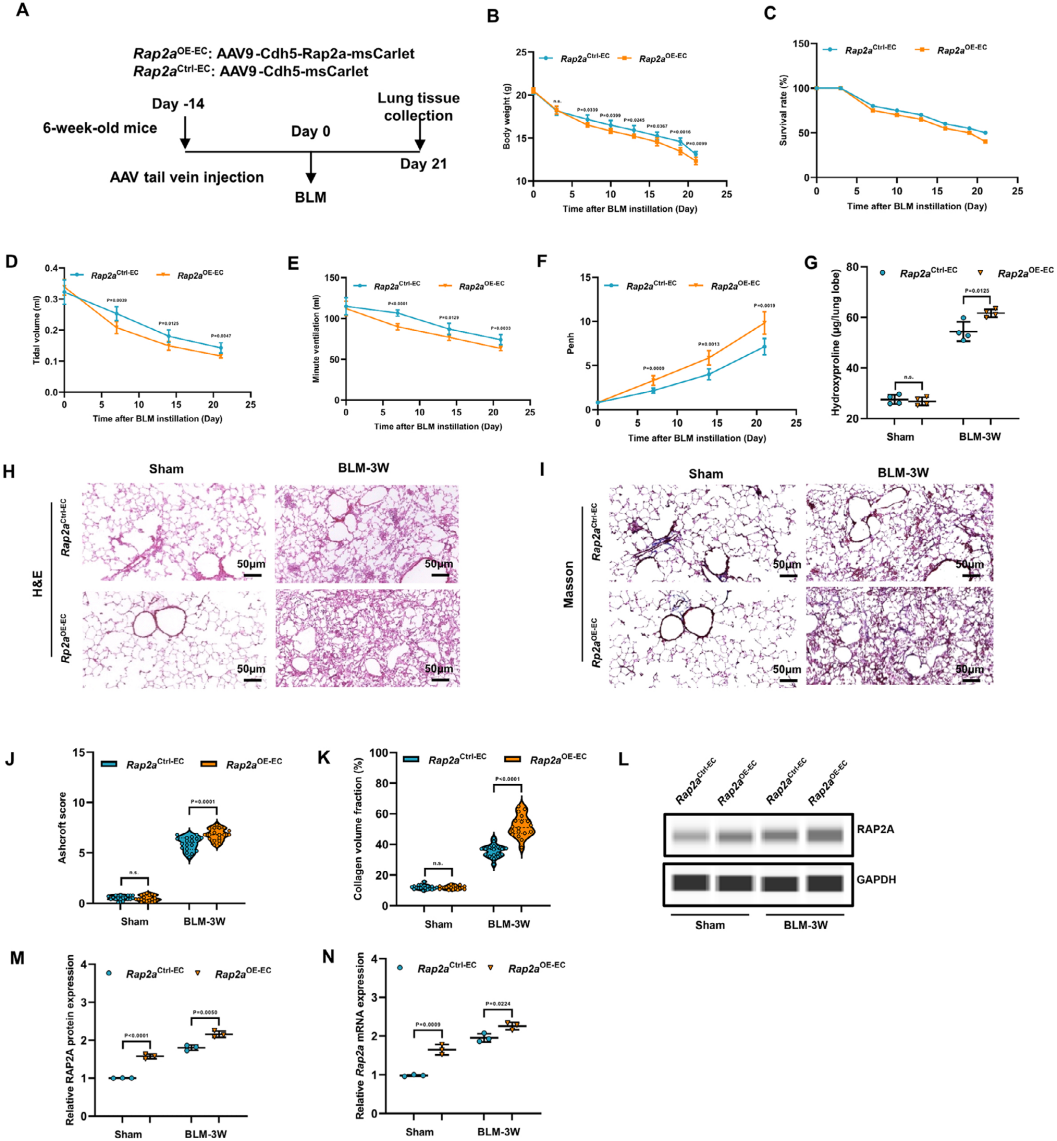
Endothelial *Rap2a* overexpression exacerbated bleomycin‐induced pulmonary fibrosis in vivo. (A) Experimental design. AAV9 vectors encoding Cdh5‐Rap2a‐mScarlet (Rap2aOE‐EC) or Cdh5‐mScarlet (*Rap2a* ^Ctrl‐EC^) were delivered via tail vein injection into wild‐type C57BL/6 mice. Two weeks after viral transduction, bleomycin (BLM) was administered intratracheally (day 0), and tissues were collected at day 21 for analyses, unless otherwise indicated. (B) Body weight change and (C) Kaplan–Meier survival curves during BLM challenge (n = 6 per group). Body weight data are presented as mean ± SEM and analyzed using two‐way repeated‐measures ANOVA with Sidak's multiple‐comparisons test; survival was analyzed using the log‐rank (Mantel–Cox) test. (D–F) Lung function parameters, including total lung volume (D), minute ventilation (E), and enhanced pause (Penh) (F) measured over the course of BLM challenge (n = 6 per group). Data are presented as mean ± SEM and analyzed using two‐way repeated‐measures ANOVA with multiple‐comparisons correction. (G) Hydroxyproline content in lung tissue at day 21 (n = 4 per group). Data are presented as mean ± SEM and analyzed using a two‐tailed unpaired *t*‐test. (H,I) Representative H&E (H) and Masson's trichrome (I) staining of lung sections at day 21 (scale bars, 50 µm). (J,K) Quantification of Ashcroft score (J) and collagen volume fraction (K) at day 21 (n = 3 per group; 6 fields per mouse). Ashcroft scores are presented as median with interquartile range (IQR) and analyzed using the Mann–Whitney test; collagen volume fraction is presented as mean ± SEM and analyzed using a two‐tailed unpaired *t*‐test. (L–N) Validation of Rap2a overexpression in lung samples collected at day 21 by Western blot (L) with densitometric quantification (M), and by qPCR for *Rap2a* mRNA (N) (n = 3 per group). Data are presented as mean ± SEM and analyzed using a two‐tailed unpaired *t*‐test. Exact *P* values are indicated.

To evaluate whether endothelial‐targeted Rap2a overexpression modulates fibrotic responses in vivo, wild‐type C57BL/6 mice were systemically transduced with AAV9‐Cdh5‐Rap2a‐mScarlet (*Rap2a* ^OE‐EC^) or the corresponding control vector AAV9‐Cdh5‐mScarlet (*Rap2a* ^Ctrl‐EC^) (Figure 5A). Two weeks after viral delivery, mice were subjected to bleomycin (BLM)‐induced lung injury. Compared with *Rap2a* ^Ctrl‐EC^ mice, *Rap2a* ^OE‐EC^ mice displayed greater body weight loss, reduced survival, and impaired lung function over the course of BLM challenge, as reflected by decreased total lung volume and minute ventilation together with increased enhanced pause (Penh) values (Figure 5B–F).

Consistent with worsened physiological outcomes, histological analyses at day 21 after BLM instillation showed more severe structural disruption and collagen deposition in *Rap2a* ^OE‐EC^ lungs than in controls, as assessed by H&E and Masson's trichrome staining (Figure 5H,I). Quantitative morphometric analyses further demonstrated increased Ashcroft scores and higher collagen volume fractions in *Rap2a* ^OE‐EC^ mice (Figure 5J,K), accompanied by elevated hydroxyproline content (Figure 5G). AAV‐mediated Rap2a overexpression was confirmed by immunoblotting and qPCR in lung samples collected at day 21 (Figure 5L–N), showing increased RAP2A protein levels and *Rap2a* mRNA expression in *Rap2a* ^OE‐EC^ mice compared with *Rap2a* ^Ctrl‐EC^ mice.

To assess whether Rap2a overexpression alone is sufficient to induce spontaneous fibrotic changes, we examined mice transduced with AAV9 vectors in the absence of BLM challenge at later time points (4–12 weeks post‐injection). As shown in Figure S4, endothelial‐targeted Rap2a overexpression did not produce overt fibrotic remodeling under baseline conditions, as indicated by preserved alveolar architecture and lack of evident collagen deposition. Together, these gain‐of‐function data support that increased endothelial Rap2a expression is associated with exacerbated fibrotic remodeling and worsened lung function in the setting of injurious stimulation.

### 2.6 Single‐Cell Transcriptomics and Cell–Cell Communication Profiling Revealed Endothelial *Rap2a* as a Pro‐Fibrotic Signaling Hub via VCAM1 Axis

**FIGURE 6:**
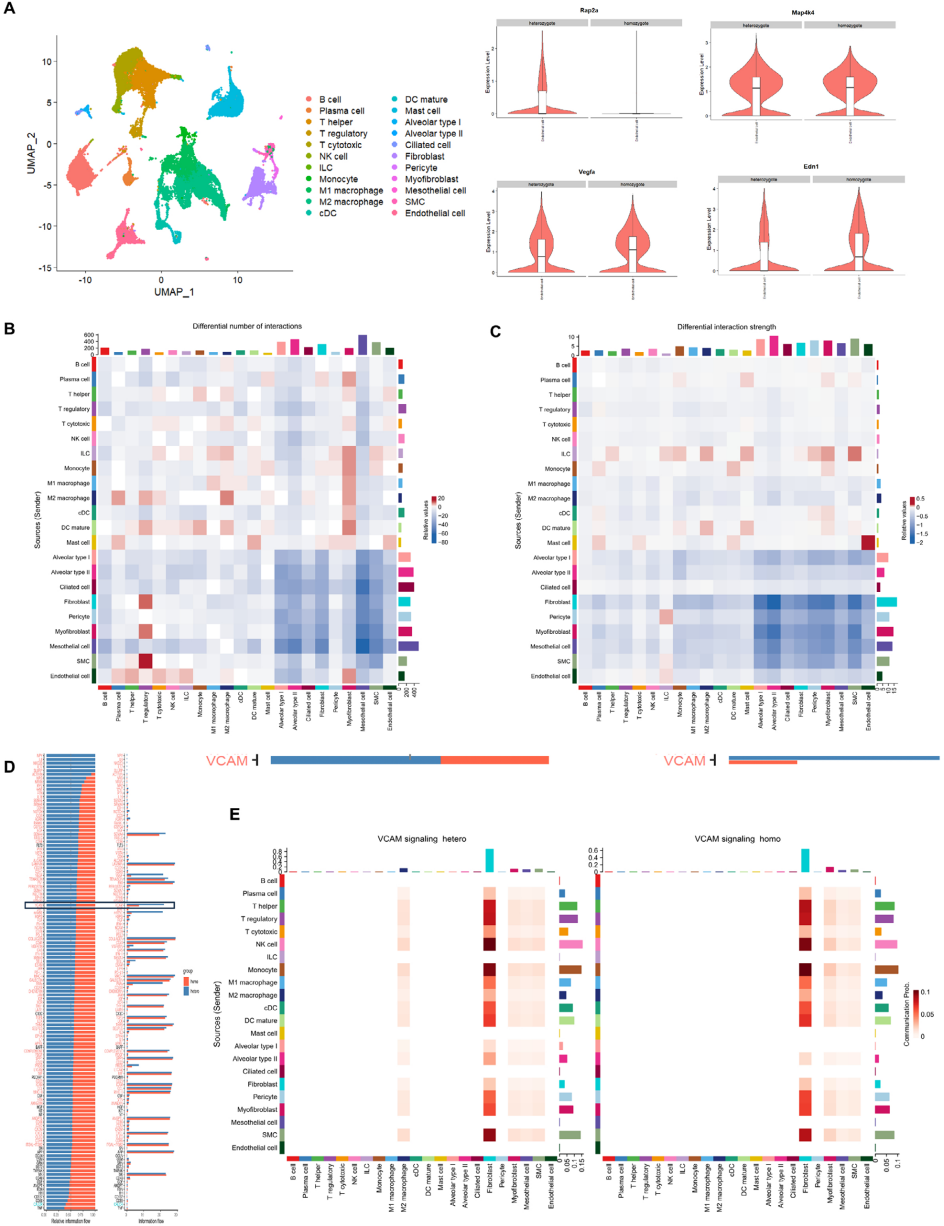
Sc RNA‐seq and intercellular signaling analysis identified endothelial *Rap2a* as a pro‐fibrotic communicator via VCAM1 pathway. (A) UMAP visualization of 20 major lung cell populations identified by scRNA‐seq (left) and violin plots showing expression of *Rap2a*, *Map4k4*, *Vcam1*, and *Edn1* across cell clusters (right). (B,C) CellChat heatmaps depicting inferred intercellular communication numbers among lung cell populations in sham (B) and BLM‐treated (C) samples. (D) Ranked information flow scores of inferred signaling pathways comparing BLM‐treated and sham lungs, highlighting increased VCAM1‐associated signaling. (E) CellChat‐predicted VCAM1 signaling networks illustrating heterotypic (left) and homotypic (right) communication patterns among sender and receiver cell populations.

To characterize the cellular distribution of Rap2a‐associated transcriptional programs during fibrotic lung injury, we performed single‐cell RNA sequencing (scRNA‐seq) on lung tissues harvested from bleomycin (BLM)‐treated mice. Unsupervised clustering resolved 20 transcriptionally distinct cell populations, including endothelial cells, fibroblasts, myofibroblasts, epithelial cells, and multiple immune cell subsets (Figure 6A, left). Analysis of gene expression patterns revealed that *Rap2a* and its reported downstream signaling component *Map4k4* were predominantly expressed in endothelial cell clusters. In addition, established fibrosis‐associated genes, including *Vcam1* and *Edn1*, showed enriched expression within endothelial and mesenchymal compartments (Figure 6A, right), suggesting a spatially restricted pro‐fibrotic transcriptional signature associated with endothelial cells.

To explore potential intercellular communication patterns linked to this transcriptional landscape, we applied CellChat analysis to infer ligand–receptor interactions among major lung cell populations. Compared with sham controls, BLM‐treated lungs exhibited increased predicted intercellular communication strength and number, with prominent changes involving endothelial cells, fibroblasts, and myeloid populations (Figure 6B,C). Among the inferred signaling pathways, VCAM1‐related interactions displayed one of the most pronounced increases in overall information flow in BLM‐treated samples relative to controls (Figure 6D).

Further network‐level analysis of the VCAM1 signaling module indicated that endothelial cells contributed substantially as both signal senders and receivers within the predicted communication network, engaging in interactions with mesenchymal and immune cell populations, including myofibroblasts, pericytes, and myeloid cells (Figure 6E). These analyses highlight endothelial cells as a central node within VCAM1‐associated communication networks during fibrotic lung injury. Collectively, the single‐cell transcriptomic and computational communication analyses suggest that endothelial Rap2a expression is associated with enhanced VCAM1‐linked intercellular signaling in experimental pulmonary fibrosis.

### 2.7 RAP2A Potentiated TNFα‐Induced MAP4K4 Activation and Endothelial Adhesive Phenotypes

**FIGURE 7:**
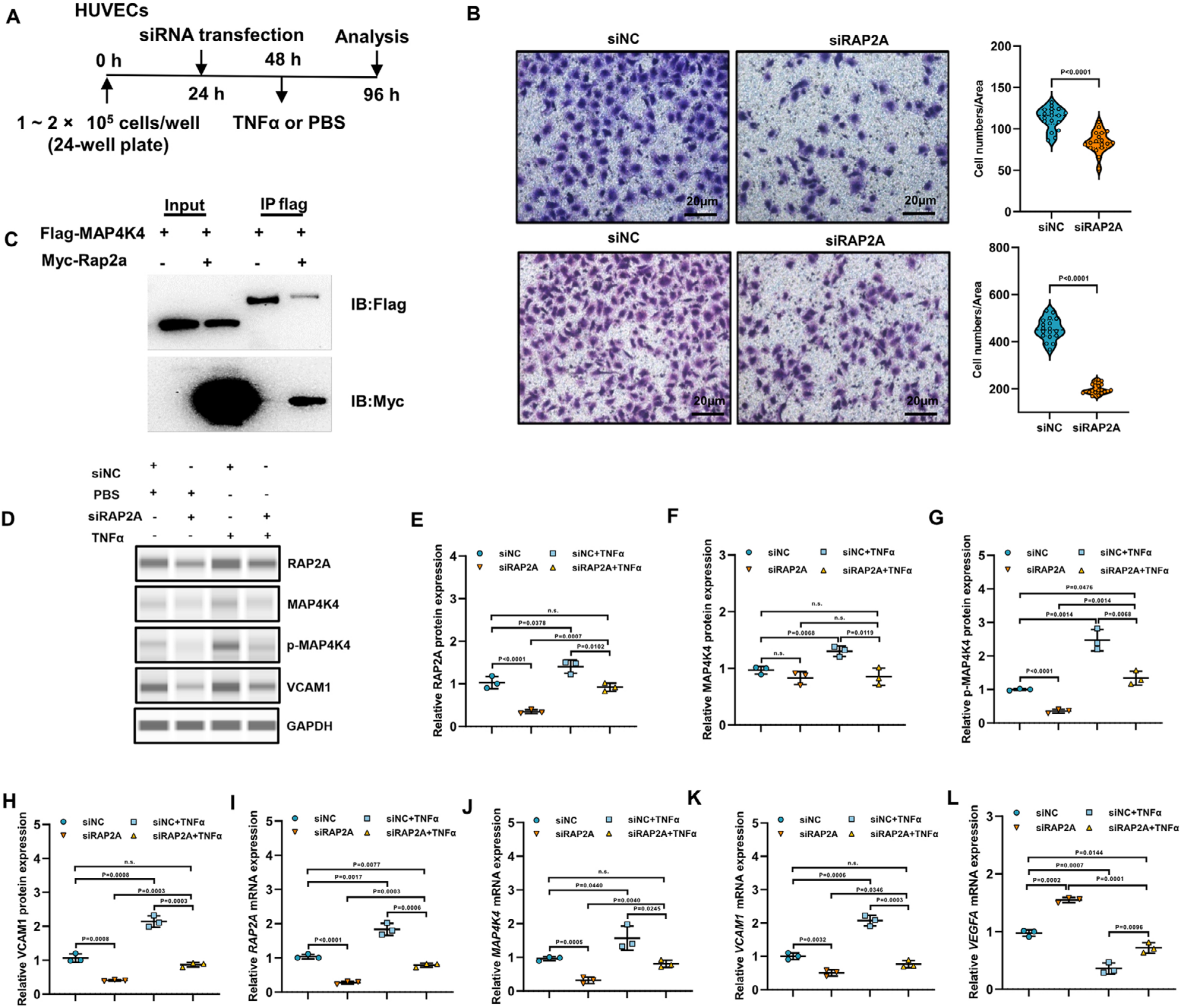
RAP2A facilitated TNF‐α–induced MAP4K4 phosphorylation and endothelial inflammatory responses. (A) Experimental timeline for siRNA‐mediated RAP2A knockdown in HUVECs followed by TNF‐α stimulation. (B) Representative images and quantification of transwell migration (upper) and monocyte adhesion (lower) assays in siNC‐ and siRAP2A‐transfected HUVECs (n = 3 independent experiments). Data are presented as mean ± SEM and analyzed using a two‐tailed unpaired *t*‐test. (C) Co‐immunoprecipitation analysis showing interaction between Flag‐tagged MAP4K4 and Myc‐tagged RAP2A in HEK293T cells. (D) Representative immunoblots showing RAP2A, total MAP4K4, phosphorylated MAP4K4 (p‐MAP4K4), and VCAM1 protein levels under the indicated conditions. (E–H) Densitometric quantification of protein expression levels is shown in (D) (n = 3 independent experiments). Data are presented as mean ± SEM and analyzed using a two‐tailed unpaired *t*‐test. (I–L) Relative mRNA expression levels of *RAP2A*, *MAP4K4*, *VCAM1*, and *VEGFA* were determined by RT‐qPCR (n = 3 independent experiments). Data are presented as mean ± SEM and analyzed using a two‐tailed unpaired *t*‐test. GAPDH was used as an internal control. Exact *P* values are indicated.

To further examine the functional relationship between RAP2A and inflammatory signaling in endothelial cells, we performed mechanistic studies in human umbilical vein endothelial cells (HUVECs). RNA interference–mediated suppression of RAP2A significantly reduced endothelial migratory capacity and attenuated monocyte adhesion to the endothelial monolayer under inflammatory conditions (Figure 7A,B), indicating an involvement of RAP2A in regulating endothelial adhesive phenotypes.

Co‐immunoprecipitation assays revealed an interaction between RAP2A and MAP4K4 in a heterologous expression system, supporting a physical association between these proteins (Figure 7C). Upon TNF‐α stimulation, RAP2A knockdown was associated with reduced MAP4K4 phosphorylation and decreased VCAM1 protein expression compared with control cells (Figure 7D–H). Consistently, transcript analyses demonstrated reduced *MAP4K4* and *VCAM1* mRNA levels following RAP2A silencing under TNF‐α exposure (Figure 7I–L).

Together, these results indicate that RAP2A contributes to TNF‐α–induced endothelial activation and pro‐adhesive responses, which are associated with MAP4K4 phosphorylation and VCAM1 upregulation. While the precise molecular mechanism governing MAP4K4 activation remains to be fully elucidated, these findings support a role for RAP2A in modulating inflammatory signaling pathways that promote endothelial activation under pro‐inflammatory conditions.

### 2.8 Erythrocyte‐Derived Nanovesicles Delivering siRap2a Achieved Pulmonary Endothelial Targeting and Attenuated Fibrosis

**FIGURE 8:**
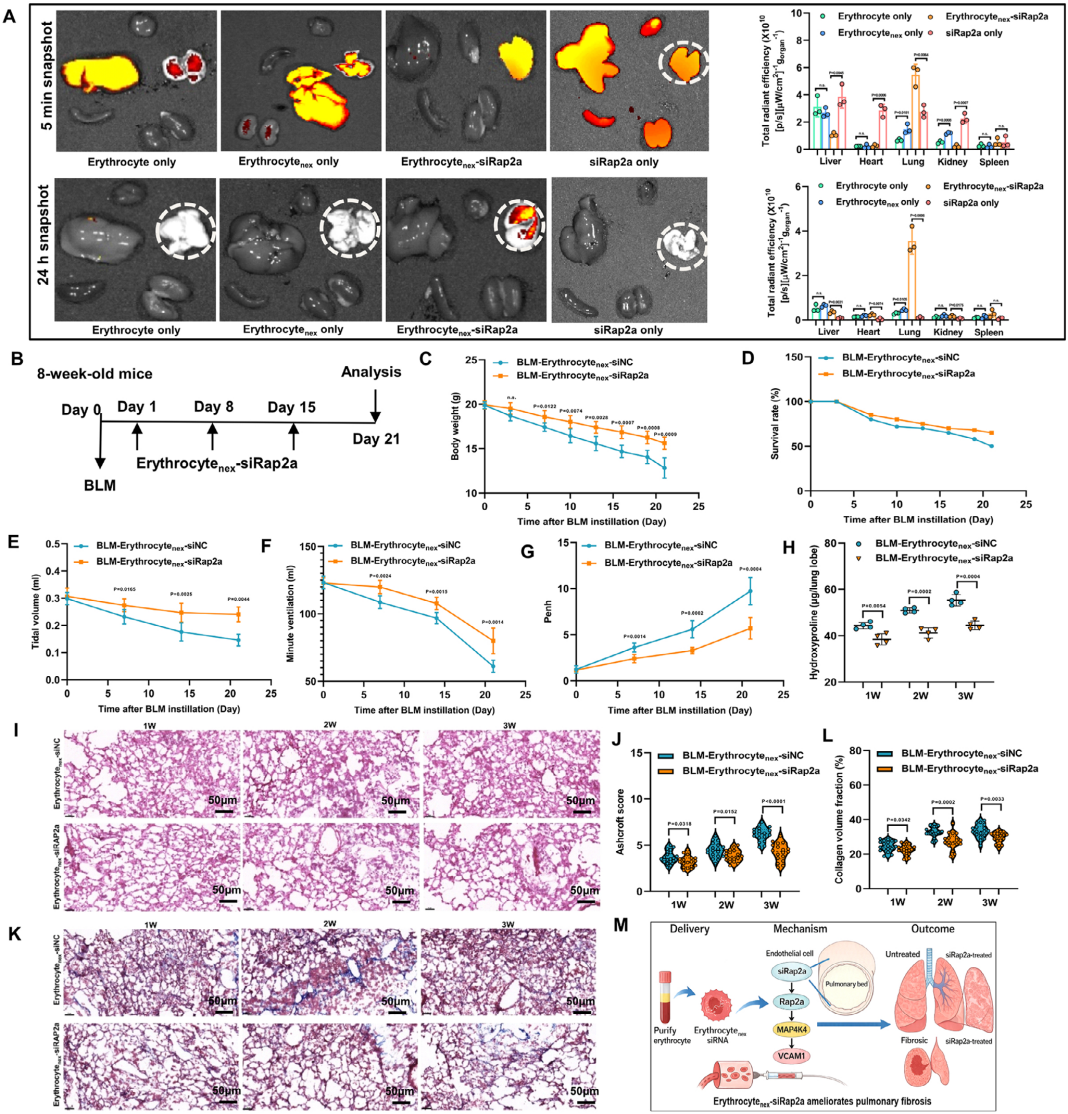
Erythrocyte_nex_‐based nanocarriers targeting endothelial *Rap2a* alleviated pulmonary fibrosis in vivo. (A) Ex vivo fluorescence imaging of major organs at 5 min and 24 h after intravenous administration of Cy5.5‐labeled siRap2a delivered by erythrocyte‐derived nanovesicles (Erythrocyte_nex_‐siRap2a), free siRap2a, or Erythrocyte_nex_‐siNC. Quantification of fluorescence intensity normalized to organ weight is shown on the right. (B) Treatment regimen schematic: mice received intratracheal bleomycin (BLM) on day 0, followed by intravenous administration of Erythrocyte_nex_‐siRap2a or control nanovesicles on days 1, 8, and 15. Endpoints were assessed up to day 21. (C–G) Effects of Erythrocyte_nex_‐siRap2a treatment on disease severity, including body weight change (C), survival (D), total lung volume (E), minute ventilation (F), and enhanced pause (Penh) (G) during BLM‐induced injury (n = 6 per group). Data are presented as mean ± SEM. Statistical analyses were performed using two‐way repeated‐measures ANOVA with Sidak's multiple‐comparisons test for longitudinal measurements and log‐rank (Mantel–Cox) test for survival. (H) Lung hydroxyproline content at day 21 after BLM challenge (n = 4 per group). Data are presented as mean ± SEM and analyzed using a two‐tailed unpaired *t*‐test. (I–K) Representative H&E (I) and Masson's trichrome staining (K) of lung sections at weeks 1, 2, and 3 after BLM administration. (J,L) Quantification of Ashcroft score (J) and collagen volume fraction (L) at weeks 1, 2, and 3 (n = 3 per group; fields per mouse as indicated). Ashcroft scores are presented as median with interquartile range and analyzed using the Mann–Whitney test; collagen volume fraction is presented as mean ± SEM and analyzed using a two‐tailed unpaired *t*‐test. Exact *P* values are indicated. (M) Schematic representation of the erythrocyte‐derived nanovesicle platform for endothelial‐targeted delivery of siRap2a.

To explore the translational therapeutic potential of Rap2a inhibition in pulmonary fibrosis, we developed erythrocyte‐derived nanovesicles encapsulating siRap2a (Erythrocyte_n__e__x_‐siRap2a). Ex vivo fluorescence biodistribution analyses demonstrated rapid pulmonary accumulation of Erythrocyte_n__e__x_‐siRap2a at 5 min after intravenous administration, with sustained lung retention observed at 24 h. This pulmonary enrichment exceeded that observed with free siRNA or control nanovesicles (Figure 8A).

Using a therapeutic regimen in which bleomycin (BLM) was administered on day 0, followed by nanovesicle injections on days 1, 8, and 15 (Figure 8B), treatment with Erythrocyte_n__e__x_‐siRap2a was associated with attenuated body weight loss and improved survival compared with control‐treated mice (Figure 8C,D). Pulmonary function was better preserved, as indicated by increased total lung volume and minute ventilation, together with reduced enhanced pause (Penh) values during the course of injury (Figure 8E–G). Consistent with functional improvement, biochemical and histological analyses revealed reduced fibrotic burden in Erythrocyte_n__e__x_‐siRap2a–treated mice. Lung hydroxyproline content was decreased, and histopathological examination showed attenuated architectural distortion and collagen deposition, with corresponding reductions in Ashcroft scores and collagen volume fractions across weeks 1, 2, and 3 after BLM challenge (Figure 8H–L).

Additional characterization of the delivery platform and its biological activity is provided in Figures S5 and S6. The nanovesicles were optimized using a polyphenol–metal coordination strategy (P/Fe^3^^+^ ratio 500:1), yielding monodisperse particles with stable hydrodynamic size and ζ‐potential. Hemolysis assays demonstrated favorable hemocompatibility, and in vitro uptake studies showed preferential internalization by CD31^+^ endothelial cells. Importantly, lung tissues from Erythrocyte_n__e__x_‐siRap2a–treated mice exhibited sustained reduction of RAP2A protein and *Rap2a* mRNA levels, consistent with effective in vivo target silencing.

Together, these data indicate that endothelial‐targeted delivery of siRap2a via erythrocyte‐derived nanovesicles is associated with reduced fibrotic remodeling and improved lung function in experimental pulmonary fibrosis.

## 3 Discussion

Pulmonary fibrosis is increasingly recognized as a disease of endothelial dysregulation, in which the vasculature functions not only as a passive target of injury but also as an active participant in immune activation and fibrotic remodeling [2, 10, 11, 12, 13]. Endothelial cells regulate leukocyte recruitment, cytokine amplification, and stromal activation, thereby shaping the trajectory of chronic lung injury [14, 15, 16]. In the present study, we investigated the role of endothelial Rap2a in experimental pulmonary fibrosis and provide evidence that Rap2a contributes to endothelial inflammatory activation and immune–vascular interactions during bleomycin‐induced lung injury. Through complementary endothelial‐enriched loss‐ and gain‐of‐function approaches, we observed that Rap2a modulates leukocyte infiltration, vascular inflammation, and extracellular matrix deposition in vivo. Moreover, systemic silencing of Rap2a using an erythrocyte_n__e__x_–based siRNA delivery strategy attenuated fibrotic remodeling and preserved pulmonary function [17, 18, 19, 20]. Collectively, these findings support a role for RAP2A as a context‐dependent modulator of endothelial inflammatory responsiveness rather than a determinant of basal vascular homeostasis [8, 21, 22].

At a mechanistic level, our data indicate that RAP2A enhances endothelial responsiveness to inflammatory stimulation through a MAP4K4–VCAM1–associated pathway. While the biochemical interaction between RAP2A and MAP4K4 has been reported previously in non‐pulmonary systems, its functional relevance in pulmonary endothelium and fibrotic disease has not been defined. In our study, RAP2A deficiency attenuated MAP4K4 phosphorylation, VCAM1 induction, and monocyte adhesion in response to TNF‐α, supporting a functional linkage between RAP2A activity and endothelial adhesive activation [23, 24, 25]. Importantly, we do not claim to resolve the molecular determinants of MAP4K4 phosphorylation or RAP2A GTP/GDP cycling, which would require dedicated biochemical and structural analyses. Instead, our data define a functional signaling framework in which RAP2A modulates inflammatory signal amplification within injured endothelium.

While earlier studies have highlighted endothelial barrier disruption, apoptosis, and aberrant angiocrine signaling as contributors to fibrogenesis [13, 26], upstream regulators that fine‐tune endothelial inflammatory reprogramming remain incompletely defined. Canonical pathways such as TGF‐β/Smad, VEGF, Notch, and WNT signaling have been extensively studied in epithelial and mesenchymal compartments [27, 28, 29], whereas the contribution of small GTPases to endothelial inflammatory adaptation has received comparatively less attention. Rap2a, a Ras‐related small GTPase, has been implicated in cytoskeletal organization, vesicle trafficking, and junctional regulation [8, 30, 31], but its involvement in fibrotic vascular activation has not been previously examined in detail. Our data extend the functional scope of Rap2a by supporting its role as a signal‐modulatory component within injured endothelium. In mice, endothelial‐specific deletion of Rap2a did not overtly perturb baseline vascular structure, yet significantly blunted TNF‐α–induced VCAM1 upregulation and monocyte adhesion, suggesting that Rap2a primarily contributes to dynamic endothelial responses under inflammatory stress rather than constitutive architectural maintenance [30, 32, 33]. By lowering endothelial sensitivity to pro‐inflammatory cues, Rap2a deficiency was associated with a microenvironment less permissive to immune cell recruitment and fibrotic progression [14, 15, 16, 34].

The functional relevance of endothelial RAP2A is further supported by gain‐of‐function experiments. Endothelium‐enriched RAP2A overexpression using AAV9‐Cdh5 did not induce spontaneous fibrosis under baseline conditions but significantly exacerbated weight loss, mortality, lung dysfunction, and histological remodeling following bleomycin challenge. These observations are consistent with a model in which RAP2A acts as a conditional amplifier of fibrogenic signaling, distinct from canonical endothelial regulators such as KLF2 or eNOS that primarily govern homeostatic or shear‐responsive phenotypes [26, 35, 36]. This stress dependency suggests a potentially favorable therapeutic window, whereby RAP2A inhibition may suppress pathological activation without broadly impairing baseline endothelial integrity.

Consistent with these functional observations, single‐cell transcriptomic profiling coupled with intercellular communication analysis revealed attenuation of endothelial–immune signaling networks in Rap2a‐deficient lungs. Transcripts encoding adhesion‐associated programs, including Vcam1, were reduced within endothelial clusters, indicating diminished endothelial receptivity to inflammatory signals [23, 24, 25, 37]. Importantly, these transcriptional changes were not accompanied by shifts in endothelial subset composition or evidence of endothelial fate transitions. Instead, Rap2a loss primarily weakened the strength of intercellular communication pathways, supporting the interpretation that Rap2a modulates signal intensity and responsiveness rather than endothelial identity per se [37, 38, 39]. These findings are consistent with a model in which RAP2A regulates the gain of endothelial signal transduction, translating inflammatory cytokine input into adhesive and immune‐recruitment outputs.

At the mechanistic level, our in vitro data support MAP4K4 as a downstream effector associated with RAP2A‐dependent endothelial activation. Knockdown of RAP2A in HUVECs attenuated MAP4K4 expression and phosphorylation, reduced VCAM1 induction, and impaired monocyte adhesion following TNF‐α stimulation [23, 24, 40]. MAP4K4 has been implicated in vascular inflammation and endothelial dysfunction in other disease contexts; for example, in diabetic cardiac microvasculature, MAP4K4 promotes mitochondrial oxidative stress and endothelial injury via regulation of Drp1 S‐nitrosylation [25]. In parallel, integrin‐mediated adhesion and mechanotransduction have emerged as key regulators of endothelial barrier dynamics and inflammatory signaling, as comprehensively reviewed by Aman et al. [36]. Although direct mechanistic coupling between Rap2a/MAP4K4 signaling and integrin‐dependent mechanotransduction was not examined in the present study, these pathways may converge in fibrotic settings characterized by altered extracellular matrix stiffness and biomechanical stress, warranting further investigation.

Rap2 family members have also been linked to Hippo/YAP pathway regulation in response to mechanical cues [8]. For instance, Rap2 has been reported to facilitate YAP activation under conditions of increased matrix stiffness via a Thrombospondin‐1–Hippo signaling axis [22]. While our data do not directly address YAP/TAZ signaling, the established involvement of Rap2 proteins in mechanosensitive pathways raises the possibility that Rap2a may intersect with broader mechanotransductive networks in fibrotic lungs, where matrix stiffening and altered mechanical stress are well documented [12, 38].

From a translational perspective, the erythrocyte_n__e__x_–siRNA delivery platform enabled efficient in vivo Rap2a silencing and produced anti‐fibrotic effects comparable to endothelial genetic deletion. This system preferentially accumulated in inflamed pulmonary tissue while exhibiting favorable biocompatibility and minimal overt toxicity. Compared with conventional viral or lipid‐based delivery approaches, erythrocyte‐derived nanovesicles offer modularity and improved tolerability, although direct comparative benchmarking was beyond the scope of this study. These findings align with emerging strategies aimed at reprogramming the vascular niche as a therapeutic avenue in fibrotic disease [18, 41, 42, 43, 44].

Several limitations of this study should be acknowledged. First, endothelial heterogeneity across vascular compartments—including capillary, arterial, venous, and lymphatic endothelia—may confer differential RAP2A sensitivity that was not fully resolved here. Second, while the RAP2A–MAP4K4–VCAM1 axis was supported by convergent genetic, transcriptional, and functional data, additional downstream pathways such as soluble adhesion molecule release, extracellular vesicle signaling, and metabolic reprogramming may also contribute to fibrotic progression. Third, upstream regulators governing Rap2a induction under fibrotic stress remain to be defined and may include inflammatory cytokines, oxidative stress, hypoxia, or biomechanical cues. Addressing these questions will require further targeted investigation.

In summary, our findings support a model in which endothelial RAP2A functions as a stress‐inducible modulator that amplifies inflammatory signaling and immune–vascular interactions during experimental pulmonary fibrosis. By integrating endothelial‐enriched genetic perturbation, single‐cell communication analysis, and functional assays, this study provides a mechanistic context and proof‐of‐concept evidence supporting RAP2A as a potential vascular‐focused therapeutic target in fibrotic lung disease.

## 4 Experimental Section

### 4.1 Human Transcriptome Dataset Analysis

Gene expression profiles of idiopathic pulmonary fibrosis (IPF) and non‐fibrotic control lung tissues were obtained from the Gene Expression Omnibus (GEO) database (accession number: GSE110147). This dataset contains bulk RNA‐sequencing data generated from human lung tissue samples. Raw count matrices were normalized and variance‐stabilized using the DESeq2 package in R. Differentially expressed genes (DEGs) were identified using an adjusted *P* value < 0.05 and |log_2_ fold change| > 1. Normalized expression levels of *RAP2A* were compared between IPF and control samples. Correlation analyses between *RAP2A* and fibrosis‐ or inflammation‐associated genes, including *MAP4K4*, *FN1*, *COL1A1*, and *VCAM1*, were performed using Pearson correlation analysis. Data visualization was conducted using the ggplot2 and pheatmap packages in R.

### 4.2 Animal Models

All animal experiments were approved by the Institutional Animal Care and Use Committee of Sichuan University and conducted in accordance with institutional and national guidelines.

For endothelial‐specific inducible knockout studies, Cdh5‐CreERT2 mice (Jackson Laboratory, #020908) were crossed with *Rap2a* ^flox/flox^ mice to generate tamoxifen‐inducible endothelial Rap2a knockout mice (*Rap2a* ^ΔEC/ΔEC^). Littermate heterozygous mice (Cdh5‐CreERT2; *Rap2a* ^flox/+^) receiving identical tamoxifen treatment served as controls (Rap2a^ΔEC/+^). Tamoxifen (Sigma, T5648) was administered intraperitoneally at 75 mg/kg/day for five consecutive days to 6–8‐week‐old mice to induce Cre‐mediated recombination.

Pulmonary fibrosis was induced by intratracheal instillation of bleomycin sulfate (3.5 mg/kg; Selleck Chemicals) in 50 µL sterile PBS under isoflurane anesthesia. Mice were euthanized at indicated time points (weeks 1, 2, or 3; corresponding to days 7, 14, and 21) after bleomycin administration for subsequent analyses.

For gain‐of‐function experiments, wild‐type C57BL/6J mice received systemic delivery of AAV9 vectors encoding mouse *Rap2a* under the control of the endothelial‐enriched Cdh5 promoter (AAV9‐Cdh5‐Rap2a) or control vectors (AAV9‐Cdh5‐mScarlet) (Obio Technology, Shanghai, China). Viruses were administered via tail vein injection at a dose of 1 × 10^1^ ^2^ viral genomes (vg) per mouse, 14 days prior to bleomycin challenge.

### 4.3 Erythrocyte‐Derived siRNA Delivery

For therapeutic intervention studies, red blood cells (RBCs) were isolated from syngeneic donor mice and processed to generate erythrocyte‐derived nanovesicles encapsulating siRNA targeting *Rap2a* (siRap2a) or negative control siRNA (siNC), as previously described. Briefly, siRNA loading was achieved via electroporation, followed by vesicle stabilization and purification.

Mice subjected to bleomycin injury received intravenous injections of erythrocyte‐derived nanovesicles (Erythrocyte_nex_‐siRap2a or control nanovesicles) on days 1, 8, and 15 after bleomycin administration. Tissue distribution and delivery efficiency were evaluated by fluorescence imaging and quantitative RT–PCR analysis of lung tissues.

### 4.4 Pulmonary Function Testing

Pulmonary function was assessed using a non‐invasive whole‐body plethysmography system (FinePointe WBP, DSI‐Buxco, USA) in conscious, unrestrained mice. Mice were individually placed into calibrated chambers and allowed to acclimate for 10 min prior to recording.

The following respiratory parameters were continuously recorded: tidal volume (TV), minute ventilation (MV), and enhanced pause (Penh). Measurements were acquired over a 5‐min period of steady‐state breathing at baseline and at days 3, 7, 10, 14, and 21 following bleomycin or sham treatment. Data were collected using FinePointe software and averaged from three reproducible recordings per animal.

### 4.5 Histological and Immunofluorescence Analysis

Lungs were perfused with PBS, inflated with 4% paraformaldehyde (PFA), and fixed overnight at 4°C. Fixed tissues were paraffin‐embedded, sectioned at 5 µm, and stained with hematoxylin and eosin (H&E) or Masson's trichrome. Fibrosis severity was evaluated using the Ashcroft scoring system by two independent investigators blinded to group allocation. Collagen deposition was semi‐quantified using ImageJ software based on the proportion of blue‐stained area in Masson‐stained sections.

For immunofluorescence analysis, frozen sections (7 µm) or paraffin sections after antigen retrieval were blocked with 5% goat serum and incubated overnight at 4°C with primary antibodies against RAP2A (Abcam), CD31 (BD Biosciences), VCAM1 (Cell Signaling Technology), F4/80 (Bio‐Rad), or α‐SMA (Sigma‐Aldrich). After washing, appropriate Alexa Fluor–conjugated secondary antibodies were applied for 1 h at room temperature. Nuclei were counterstained with DAPI. Images were acquired using Leica or Zeiss confocal microscopes, and quantification was performed on 3–6 randomly selected high‐power fields per mouse using ImageJ.

### 4.6 Hydroxyproline Quantification

Pulmonary collagen content was quantified by measuring hydroxyproline levels using a colorimetric assay adapted from established protocols. Briefly, 20–30 mg of left lung tissue was hydrolyzed in 500 µL of 6 N HCl at 110°C for 16 h in sealed glass tubes. After hydrolysis, samples were neutralized with NaOH and incubated with chloramine‐T reagent (final concentration 0.05 m) at room temperature for 20 min, followed by reaction with Ehrlich's reagent (1 m *p*‐dimethylaminobenzaldehyde in perchloric acid) at 65°C for 20 min. Absorbance was measured at 560 nm using a microplate reader. A standard curve was generated using trans‐4‐hydroxy‐L‐proline (Sigma‐Aldrich), and total hydroxyproline content was calculated and normalized to lung tissue wet weight.

### 4.7 Bronchoalveolar Lavage Fluid (BALF) Collection and Cytokine Analysis

Bronchoalveolar lavage was performed by instilling and retrieving 1 mL of ice‐cold PBS into the lungs via a tracheal cannula. BALF samples were centrifuged at 400 × *g* for 10 min at 4°C to separate cells from supernatant. Total cell numbers were determined using a hemocytometer, and differential cell counts were performed on cytospin preparations stained with Wright–Giemsa. BALF supernatants were collected and stored at −80°C until analysis.

Concentrations of TNF‐α and IL‐6 in BALF were measured using commercially available ELISA kits (R&D Systems) according to the manufacturer's instructions. All samples were assayed in duplicate, and cytokine concentrations were calculated from standard curves.

### 4.8 Flow Cytometry

Lung tissues were harvested, minced, and enzymatically digested in RPMI‐1640 medium containing collagenase D (2 mg/mL) and DNase I (100 µg/mL) at 37°C for 45 min. Cell suspensions were filtered through a 70‐µm nylon mesh and subjected to red blood cell lysis using ACK buffer. After washing, cells were incubated with anti‐mouse CD16/32 antibody (BioLegend) to block Fc receptors and stained with fluorochrome‐conjugated antibodies against CD45 (clone 30‐F11, BioLegend) and CD31 (clone MEC13.3, BioLegend).

Dead cells were excluded using a fixable viability dye (eBioscience). Flow cytometric acquisition was performed on a Cytek Aurora spectral cytometer, and data were analyzed using FlowJo software (v10). Endothelial cells were defined as CD45^−^CD31^+^, and hematopoietic lineage cells were defined as CD45^+^CD31^−^. Population frequencies were calculated as percentages of total live singlet cells.

### 4.9 Cell Culture and Treatment

Primary human umbilical vein endothelial cells (HUVECs) were obtained from ScienCell Research Laboratories and cultured in Endothelial Cell Medium (ECM; ScienCell) supplemented with 5% fetal bovine serum (FBS), 1% endothelial cell growth supplement, and 1% penicillin–streptomycin. Cells were maintained at 37°C in a humidified 5% CO_2_ incubator and used between passages 3–6.

For gene silencing experiments, HUVECs were transfected with siRNA targeting human *RAP2A* (siRAP2A) or non‐targeting control siRNA (siNC) (GenePharma) using Lipofectamine RNAiMAX (Thermo Fisher Scientific). Transfections were performed at 50–70% confluency in Opti‐MEM medium, and cells were harvested 48 h later for downstream analyses.

### 4.10 Migration and Monocyte Adhesion Assays

For transwell migration assays, siRNA‐transfected HUVECs (1 × 10^5^ cells) were seeded into the upper chambers of transwell inserts (8‐µm pore size, Corning) in serum‐free medium, with ECM containing 10% FBS placed in the lower chambers as a chemoattractant. After 16 h, migrated cells on the lower membrane surface were fixed, stained with crystal violet, and quantified under a light microscope.

For monocyte adhesion assays, HUVECs were seeded in 24‐well plates and stimulated with TNF‐α (10 ng/mL) or PBS for 6 h. CFSE‐labeled THP‐1 monocytes (1 × 10^6^ cells/mL) were added and co‐incubated for 30 min at 37°C. Non‐adherent cells were removed by gentle washing, and adherent monocytes were imaged and quantified using ImageJ.

### 4.11 Western Blot and Quantitative RT‐PCR

Protein expression was analyzed using the WES automated capillary‐based immunoassay system (ProteinSimple). Lung tissues or cultured cells were lysed in RIPA buffer supplemented with protease and phosphatase inhibitors, and protein concentrations were determined by BCA assay. For each sample, 0.8 µg of total protein was loaded per capillary according to the manufacturer's protocol.

Primary antibodies included anti‐RAP2A (Abcam), anti‐VCAM1 (Cell Signaling Technology), anti‐MAP4K4, anti‐phospho‐MAP4K4 (Ser791) (Cell Signaling Technology), and anti‐GAPDH (Proteintech). Protein expression levels were quantified using Compass software and normalized to GAPDH.

Total RNA was extracted using TRIzol reagent (Invitrogen). One microgram of RNA was reverse‐transcribed using a cDNA synthesis kit (Takara). Quantitative PCR was performed using SYBR Green Master Mix (Thermo Fisher Scientific) on a LightCycler 480 system (Roche). Gene expression was normalized to GAPDH using the 2^−^ΔΔCt method. Primer sequences are listed in Table S1.

### 4.12 Co‐Immunoprecipitation (Co‐IP)

HUVECs were lysed in ice‐cold IP lysis buffer (50 mm Tris‐HCl, pH 7.4; 150 mm NaCl; 1% NP‐40) containing protease and phosphatase inhibitors. Lysates were cleared by centrifugation and pre‐incubated with protein A/G magnetic beads for 1 h at 4°C to reduce nonspecific binding. Supernatants were incubated overnight at 4°C with 2 µg of anti‐RAP2A (Proteintech, Cat#11421‐1‐AP) or control antibodies, followed by incubation with fresh beads for 2 h.

Immunoprecipitated complexes were eluted in SDS sample buffer, resolved by SDS–PAGE, transferred to PVDF membranes, and analyzed by standard immunoblotting with HRP‐conjugated secondary antibodies and chemiluminescent detection.

### 4.13 Single‐Cell RNA Sequencing and Cell‐Cell Communication Analysis

Single‐cell suspensions were prepared from mouse lungs harvested at day 21 after bleomycin challenge. Lungs were perfused, minced, and digested with collagenase I (1 mg/mL) and DNase I (50 µg/mL) for 30 min at 37°C. After filtration and erythrocyte lysis, viable cells were resuspended in PBS containing 0.04% BSA and processed using the 10x Genomics Chromium platform.

Libraries were generated using the Chromium Single Cell 3′ v3 kit and sequenced on an Illumina NovaSeq 6000 system. Raw sequencing data were processed using Cell Ranger (v6.0.1) and aligned to the mm10 reference genome.

Downstream analyses, including quality control, normalization, dimensionality reduction (PCA, UMAP), and clustering, were performed using Seurat (v4.1.0). Cell types were annotated based on canonical marker genes. Cell–cell communication inference was conducted using CellChat (v1.5.0) to predict ligand–receptor interactions among major lung cell populations.

### 4.14 Statistical Analysis

All quantitative data are presented as mean ± SEM unless otherwise specified. Comparisons between two groups were performed using unpaired two‐tailed Student's *t*‐tests. For comparisons involving multiple groups or time points, one‐way or two‐way ANOVA followed by Bonferroni's post hoc test was applied as appropriate. Survival curves were analyzed using the log‐rank (Mantel–Cox) test. Exact *P* values are reported in figures where applicable. Statistical analyses were performed using GraphPad Prism 9.5.1, and *P* < 0.05 was considered statistically significant.

## Author Contributions

Y.L, Y.H, and B.D conceptualized the study; Y.L, Y.Q, Y.H, B.D, X.Z, P.Y, K.Z, and J.G designed the experiments; X.Z, P.Y, K.Z, G.D, Y.Z, S.L, X.L, Y.Z, S.J, and Y.Q performed experiments; X.Z, P.Y, Y.Z, S.L, X.L, Y.Z, S.J, and Y.Q analyzed data; X.Z, B.D, Y.H, and Y.L wrote or edited the manuscript.

## Ethics Approvals

All animal experiments were reviewed and approved by the Experimental Animal Management and Ethics Committee of West China Second University Hospital, Sichuan University (Approval No. 2024149), and were conducted in accordance with institutional guidelines for the care and use of laboratory animals.

## Conflicts of Interest

The authors declare no conflict of interest.

## Supporting information




**Supporting File**: advs74338‐sup‐0001‐SuppMat.docx.

## Data Availability

The data that support the findings of this study are available from the corresponding author upon reasonable request. Bulk RNA‐seq datasets analyzed in this study were obtained from the Gene Expression Omnibus (GEO) under accession number GSE110147. Single‐cell RNA‐seq data were generated for mechanistic investigation and are not publicly deposited at this stage due to data volume and ongoing follow‐up analyses, but will be made available upon reasonable request. No new human or clinical trial datasets were generated in this study.
