# Supplementary material for: Inactivation Rap2a in Endothelial Cell Prevents Pulmonary Fibrosis by Regulating Immune Microenvironment Through MAP4K4‐VCAM1 Signaling
Source: Adv Sci (Weinh). 2026 Feb 11;13(22):e19892. doi: 10.1002/advs.202519892 (PMC13088280; doi:10.1002/advs.202519892)
Supplement: Supplementary file 1 — Supporting File: advs74338‐sup‐0001‐SuppMat.docx. [file ADVS-13-e19892-s001.docx]

# **Supporting information**

**Inactivation *Rap2a* in endothelial cell prevents pulmonary fibrosis by regulating immune microenvironment through MAP4K4-VCAM1 signaling**

Running title: *Rap2a* and pulmonary fibrosis

Xiaolan Zheng^1^#, Peng Yue^1^#, Kaiyu Zhou^1^#, Guidong Gong^2^, Yue Zhang^1^, Sha Lin^1^, Xu Liu^1^, Yanjiang Zheng^1^, Siyuan Jing^1^, Junling Guo^3^, Yan Qi^4^†, Bi-Sen Ding^5^†, Yimin Hua^1^†, Yifei Li^1,6^†,

1. Key Laboratory of Birth Defects and Related Diseases of Women and Children of MOE, Department of Pediatrics, West China Second University Hospital, Sichuan University, Chengdu, Sichuan 610041, China.

2. Institute of Cardiovascular Surgery, The Second Affiliated Hospital, Army Medical University, Chongqing, 400037, China.

3. BMI Center for Biomass Materials and Nanointerfaces, College of Biomass Science and Engineering, Sichuan University, Chengdu, Sichuan 610065, China.

4. State Key Laboratory of Biocatalysis and Enzyme Engineering, School of Life Science, Hubei University, Wuhan, Hubei 430062, China.

5. Key Lab of Birth Defects and Related Diseases of Women and Children of MOE, State Key Lab of Biotherapy, State Key Laboratory of Respiratory Health and Multimorbidity, West China School of Basic Medical Sciences & Forensic Medicine, West China Second University Hospital, Sichuan University, Chengdu, Sichuan 610041, China.

6. Department of Pediatric Cardiovascular, The Second Hospital & Clinical Medical School, Lanzhou University, Lanzhou, Gansu 730030, China.

# These authors contributed equally to this work.

**†Correspondence to:**

Yifei Li, MD, Key Laboratory of Birth Defects and Related Diseases of Women and Children of MOE, Department of Pediatrics, West China Second University Hospital, Sichuan University, 20 3rd Section, Renmin S.Rd., Chengdu, Sichuan, 610041. Email: [liyfwcsh@scu.edu.cn](mailto:liyfwcsh@scu.edu.cn). (Leading contact)

Yimin Hua, MD, Key Laboratory of Birth Defects and Related Diseases of Women and Children of MOE, Department of Pediatrics, West China Second University Hospital, Sichuan University, 20 3rd Section, Renmin S.Rd., Chengdu, Sichuan, 610041. Email: nathan_hua@163.com;

Bi-Sen Ding, PhD, Key Lab of Birth Defects and Related Diseases of Women and Children of MOE, State Key Lab of Biotherapy, State Key Laboratory of Respiratory Health and Multimorbidity, West China School of Basic Medical Sciences & Forensic Medicine, West China Second University Hospital, Sichuan University, 20 3rd Section, Renmin S.Rd., Chengdu, Sichuan 610041, China. Email: dingbisen@scu.edu.cn;

Yan Qi, PhD, State Key Laboratory of Biocatalysis and Enzyme Engineering, School of Life Science, Hubei University, 318 Youyi Ave, Wuhan, Hubei, 430062, China. Email: qiyan@hubu.edu.cn.


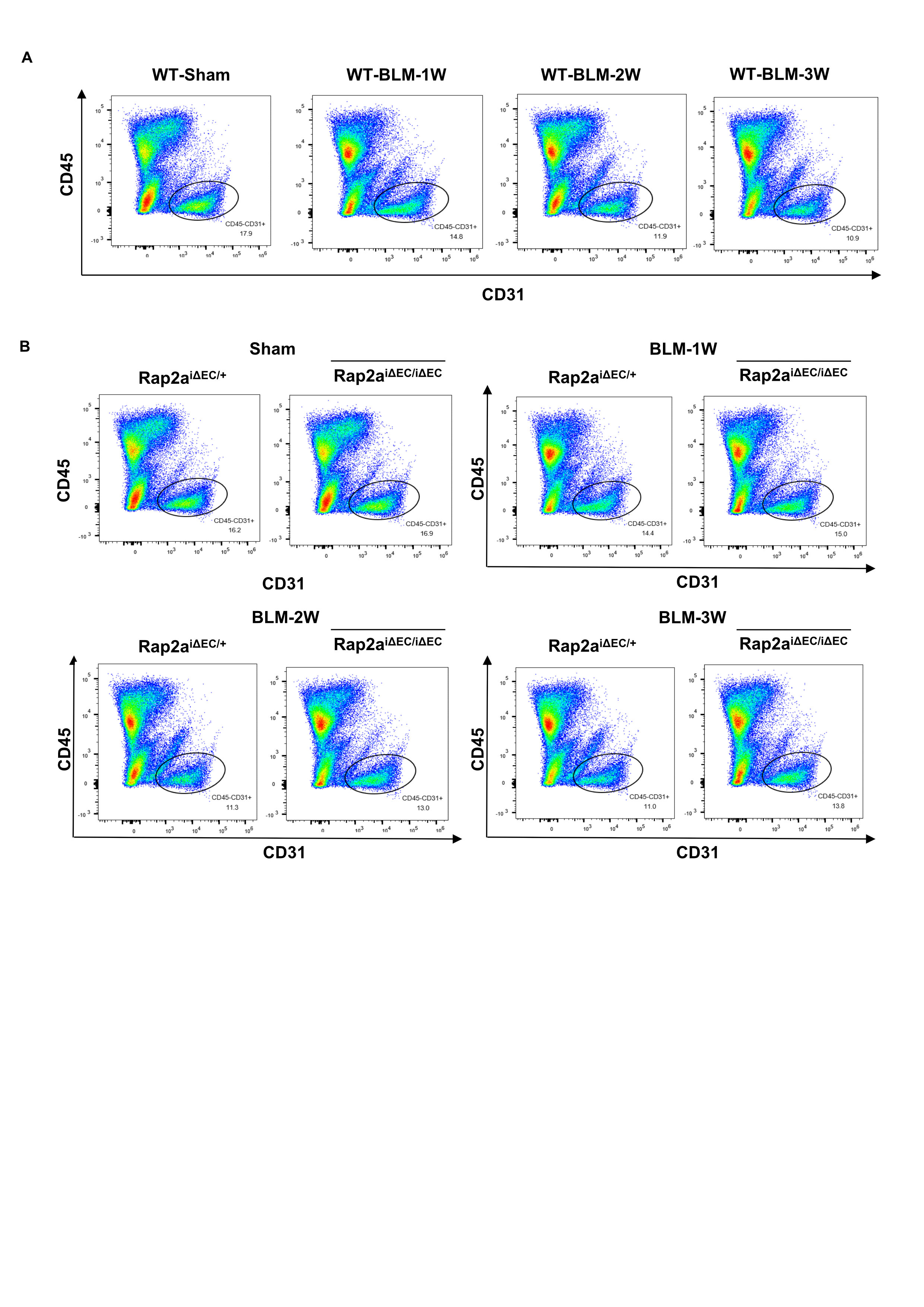


**Supplementary Figure S1. Flow cytometric analysis of endothelial cell population dynamics during BLM-induced lung fibrosis.** (A) Representative flow cytometry plots from wild-type (WT) mice showing the proportion of CD31⁺CD45⁻ endothelial cells in lung single-cell suspensions at baseline (sham) and at 1, 2, and 3 weeks after bleomycin (BLM) administration. (B) Flow cytometric comparison of CD31⁺CD45⁻ endothelial cell frequencies between *Rap2a*^ΔEC/+^ and *Rap2a*^ΔEC/ΔEC^ mice at 1, 2, and 3 weeks after BLM challenge. Quantification shows a progressive reduction of endothelial cells during fibrotic progression, which was attenuated in mice with endothelial Rap2a deletion. Data are presented as mean ± SEM. Exact *P* values are indicated.


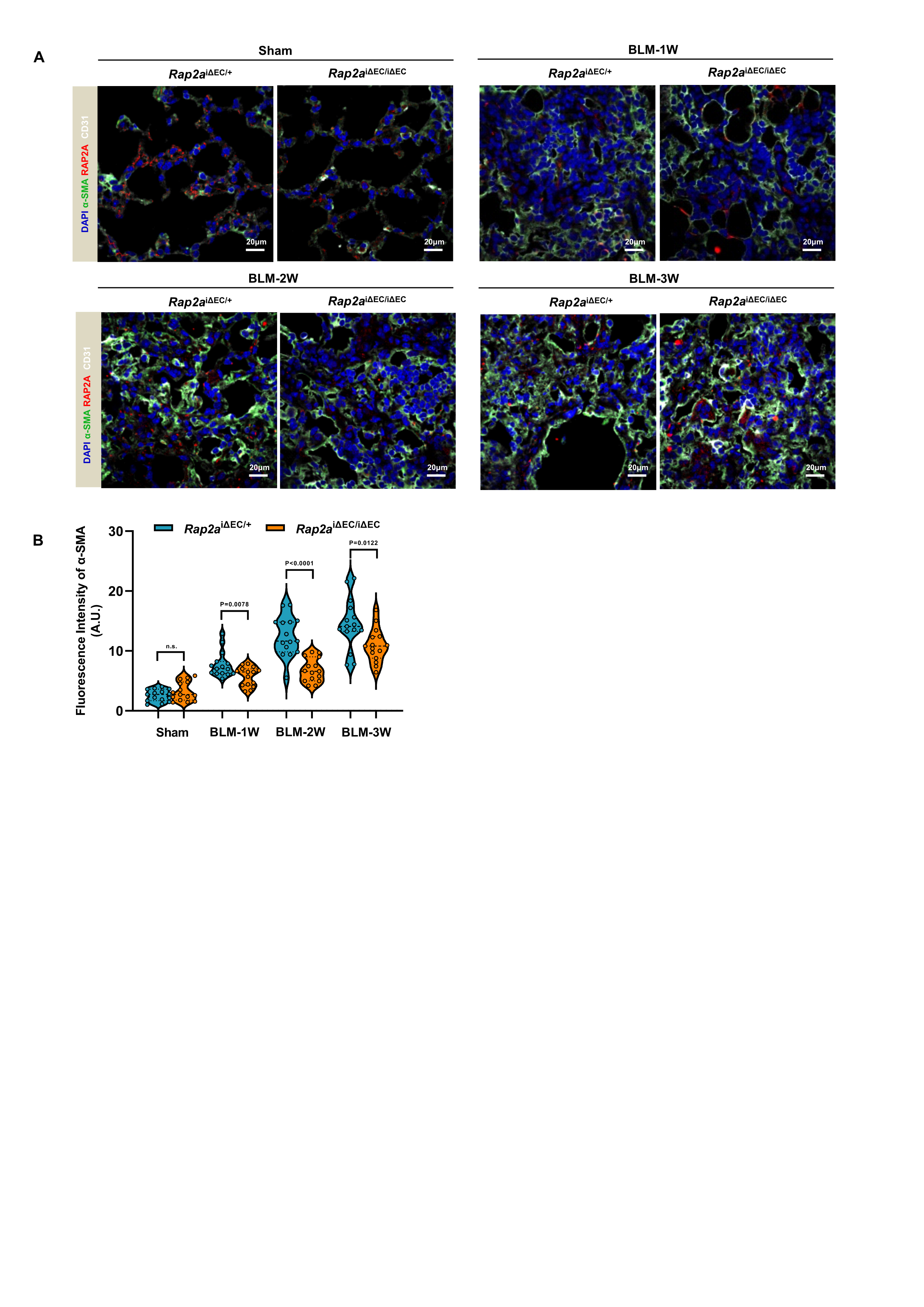


**Supplementary Figure S2. Endothelial Rap2a deletion reduced myofibroblast activation during fibrotic progression.** (A) Representative immunofluorescence staining of lung sections from *Rap2a*^ΔEC/+^ and *Rap2a*^ΔEC/ΔEC^ mice at indicated time points after BLM administration, showing α-SMA (red), CD31 (green), and nuclei (DAPI, blue). (B) Quantification of α-SMA fluorescence intensity presented as violin plots demonstrates reduced myofibroblast-associated α-SMA signal in *Rap2a*^ΔEC/ΔEC^ lungs at 1–3 weeks after BLM challenge. Exact P values are indicated.


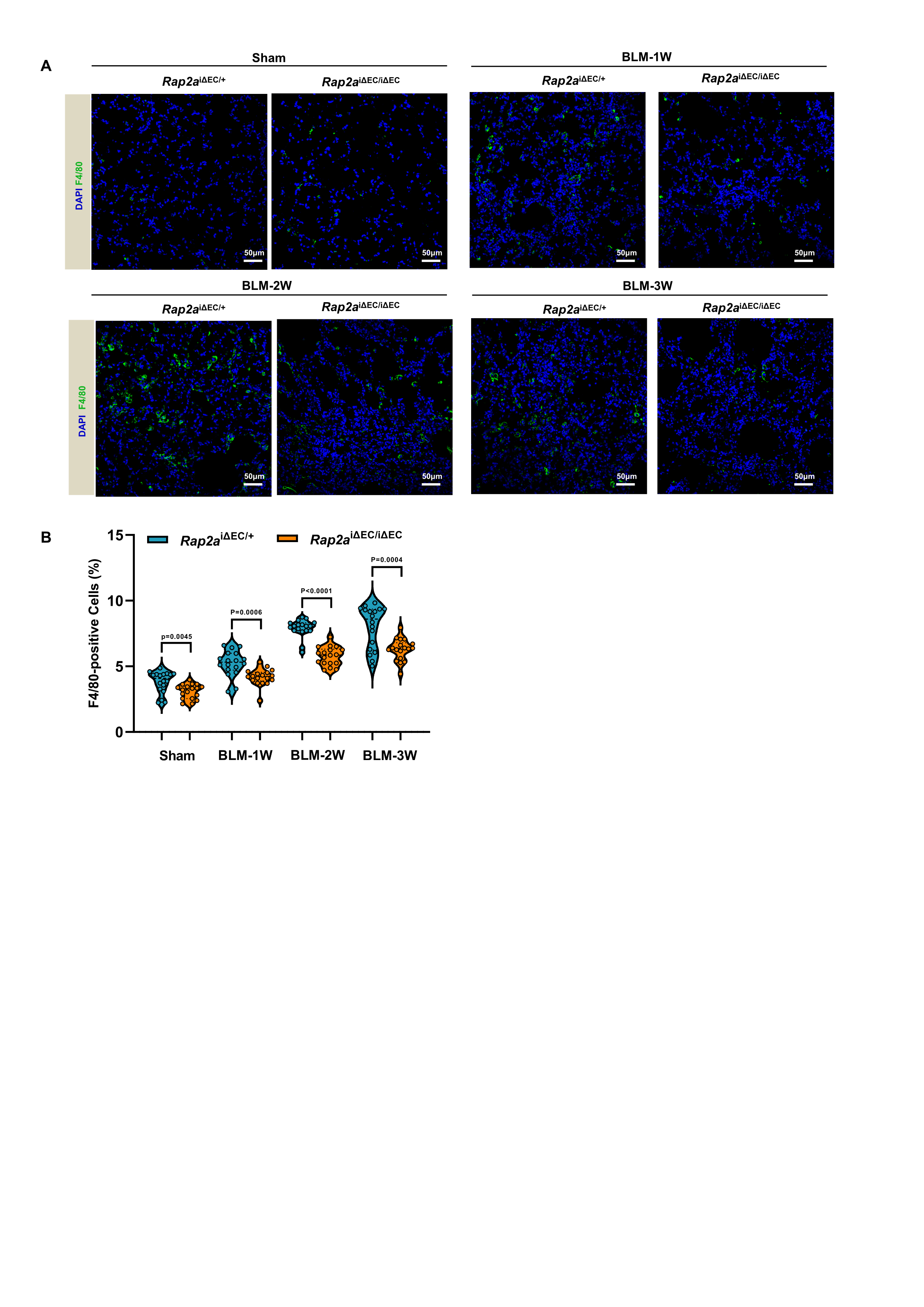


**Supplementary Figure S3. Endothelial *Rap2a* deletion reduced pulmonary macrophage accumulation.** (A) Representative immunofluorescence staining for F4/80 (green) in lung sections from *Rap2a*^ΔEC/+^ and *Rap2a*^ΔEC/ΔEC^ mice under sham and BLM-treated conditions. Nuclei were counterstained with DAPI (blue). (B) Quantitative analysis of F4/80⁺ macrophage infiltration shows reduced macrophage accumulation in Rap2a-deficient lungs, particularly at 2 and 3 weeks after BLM administration. Data are presented as mean ± SEM. Exact *P* values are indicated.


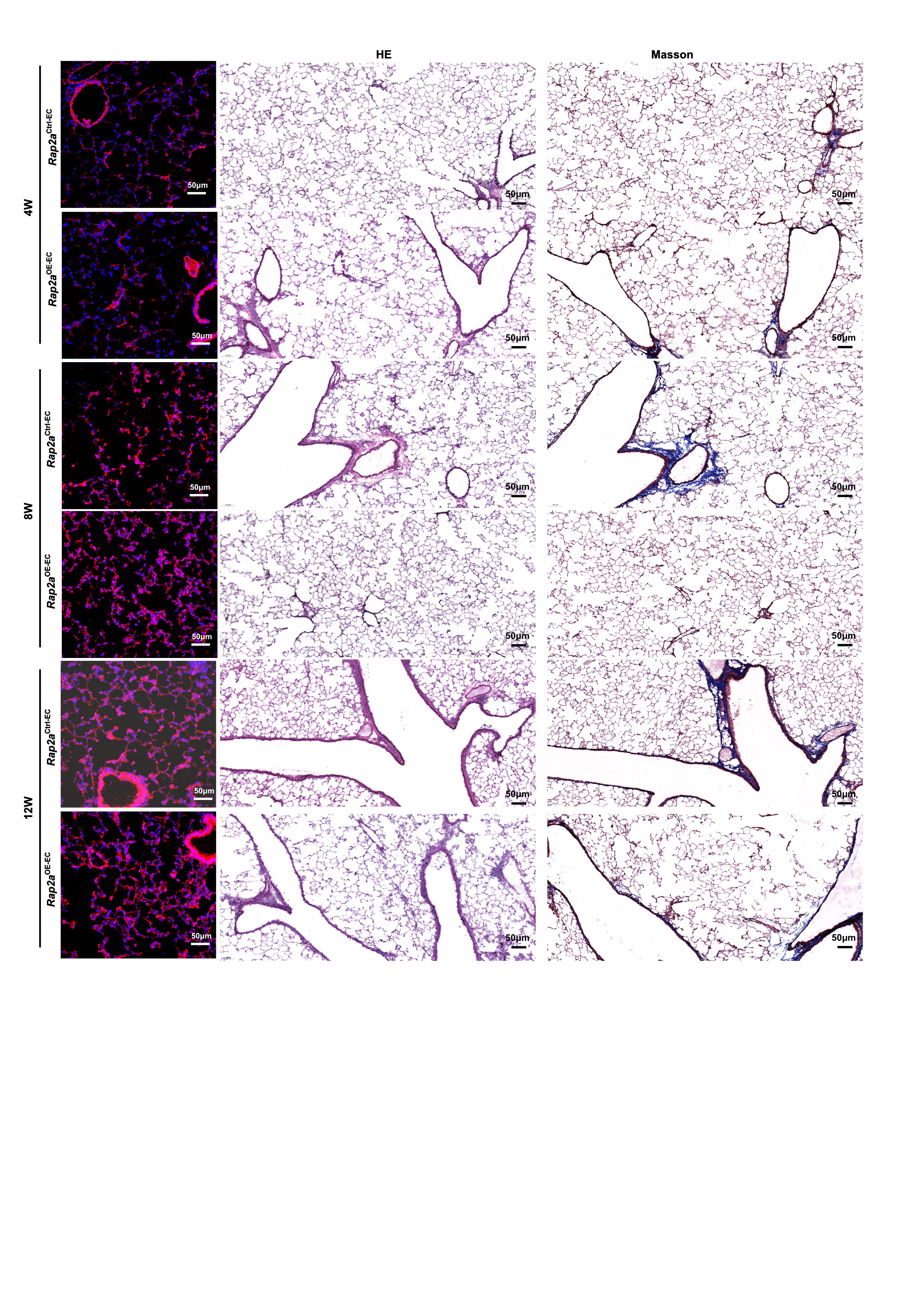


**Supplementary Figure S4. Sustained endothelial expression of RAP2A in transgenic Rap2a^EC-OE^ mice prolonged fibrosis.** Immunofluorescence staining (left) confirms persistent endothelial Rap2a expression in lungs from AAV9-Cdh5-Rap2a–treated mice compared with control mice at 4, 8, and 12 weeks after viral delivery in the absence of BLM challenge. Representative H&E (middle) and Masson’s trichrome (right) staining show preserved alveolar architecture and absence of overt collagen deposition across time points.


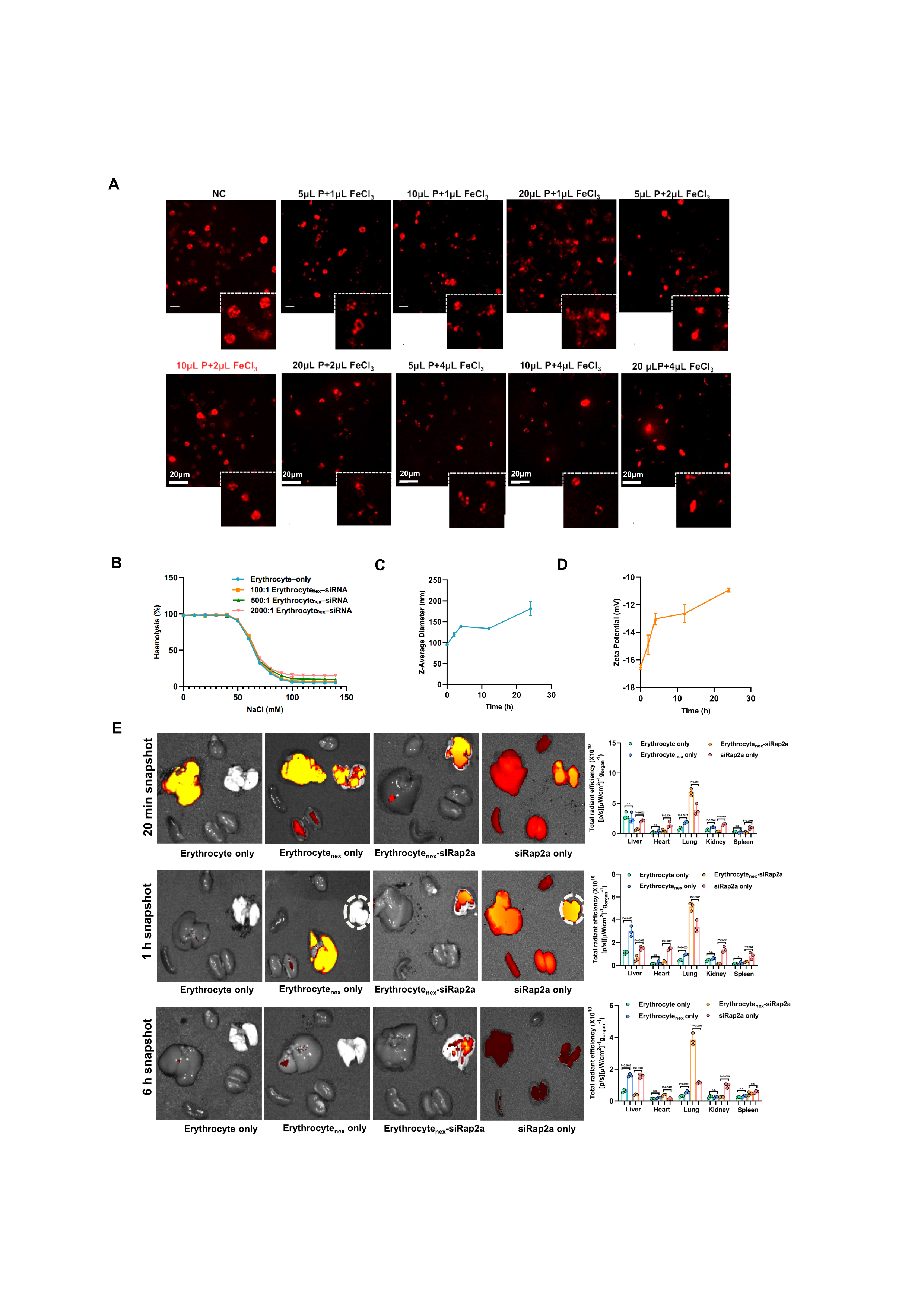


**Supplementary Figure S5. Optimization and characterization of erythrocyte_nex_-siRap2a nanocarriers.** (A) Representative fluorescence images of nanocomplexes assembled using varying polyphenol (P) and Fe³⁺ concentrations. The 10 μL P + 2 μL Fe³⁺ formulation (P/Fe³⁺ ratio 500:1) yielded uniform nanovesicles. (B) Hemolysis assays demonstrating minimal erythrocyte lysis under tested conditions. (C–D) Time-dependent measurements of hydrodynamic size (C) and zeta potential (D), indicating formation of stable, negatively charged nanovesicles. (E) Ex vivo fluorescence imaging of major organs at 20 min, 1 h, and 6 h after intravenous administration showing preferential pulmonary accumulation of erythrocyte_nex_-siRap2a compared with control groups. Quantification of fluorescence intensity normalized to organ weight is shown on the right. Data are presented as mean ± SEM. Exact *P* values are indicated.


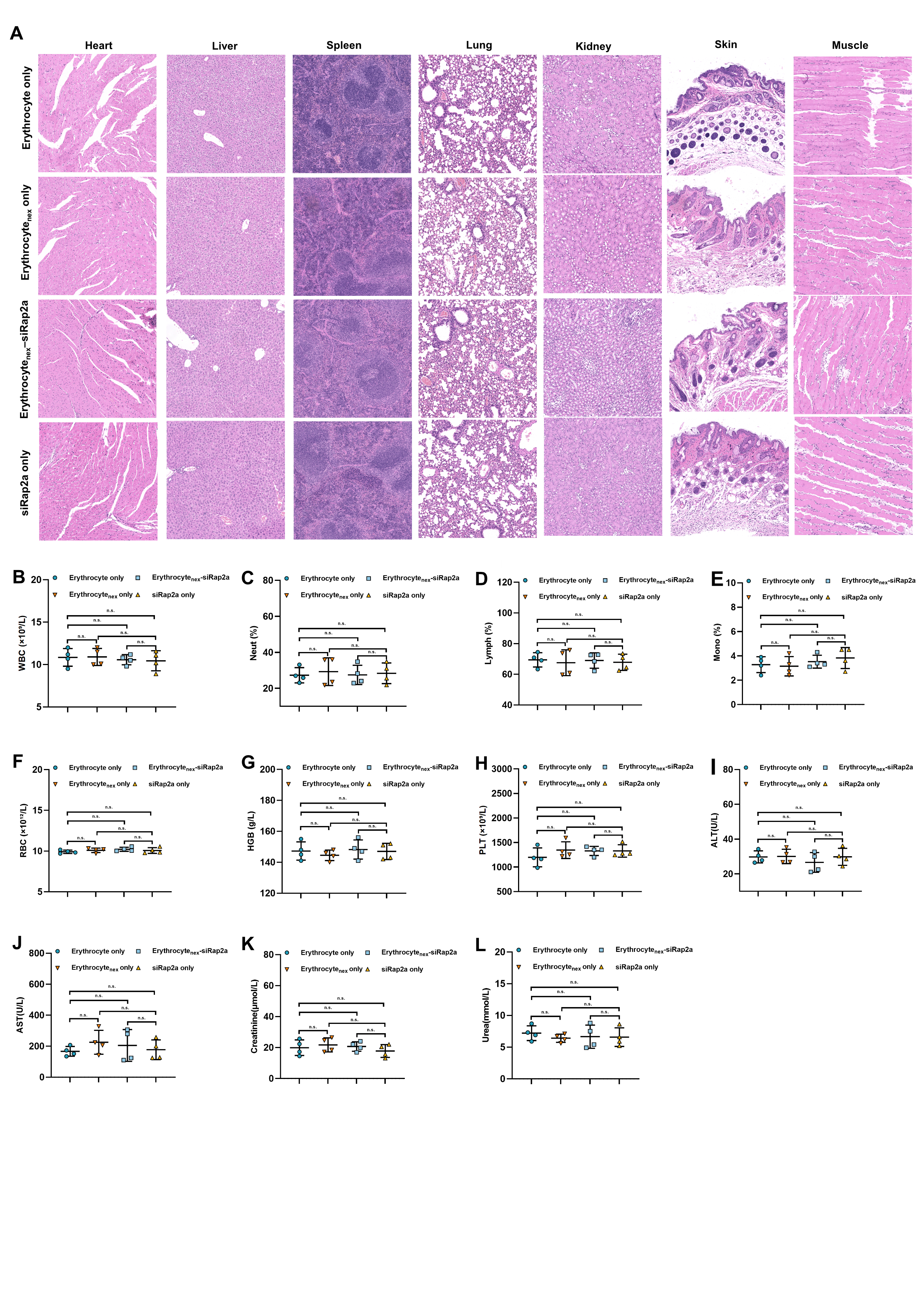


**Supplementary Figure S6. Systemic safety evaluation of erythrocyte_nex_-siRap2a** treatment in vivo**.** (A) Representative H&E staining of major organs, including heart, liver, spleen, lung, kidney, skin, and skeletal muscle, from mice treated with erythrocytes alone, erythrocyte_nex_ alone, erythrocyte_nex_-siRap2a, or free siRap2a. No overt histopathological abnormalities were observed. (B–E) Peripheral blood leukocyte parameters, including total white blood cell counts (B), neutrophil percentage (C), lymphocyte percentage (D), and monocyte percentage (E). (F–H) Red blood cell–related parameters, including red blood cell count (F), hemoglobin concentration (G), and platelet count (H). (I–J) Serum liver function markers alanine aminotransferase (ALT) (I) and aspartate aminotransferase (AST) (J). (K–L) Renal function indicators including serum creatinine (K) and blood urea nitrogen (L). Data are presented as mean ± SEM and analyzed using one-way ANOVA with appropriate post hoc tests. *n* = 4 mice per group. Exact *P* values are indicated.


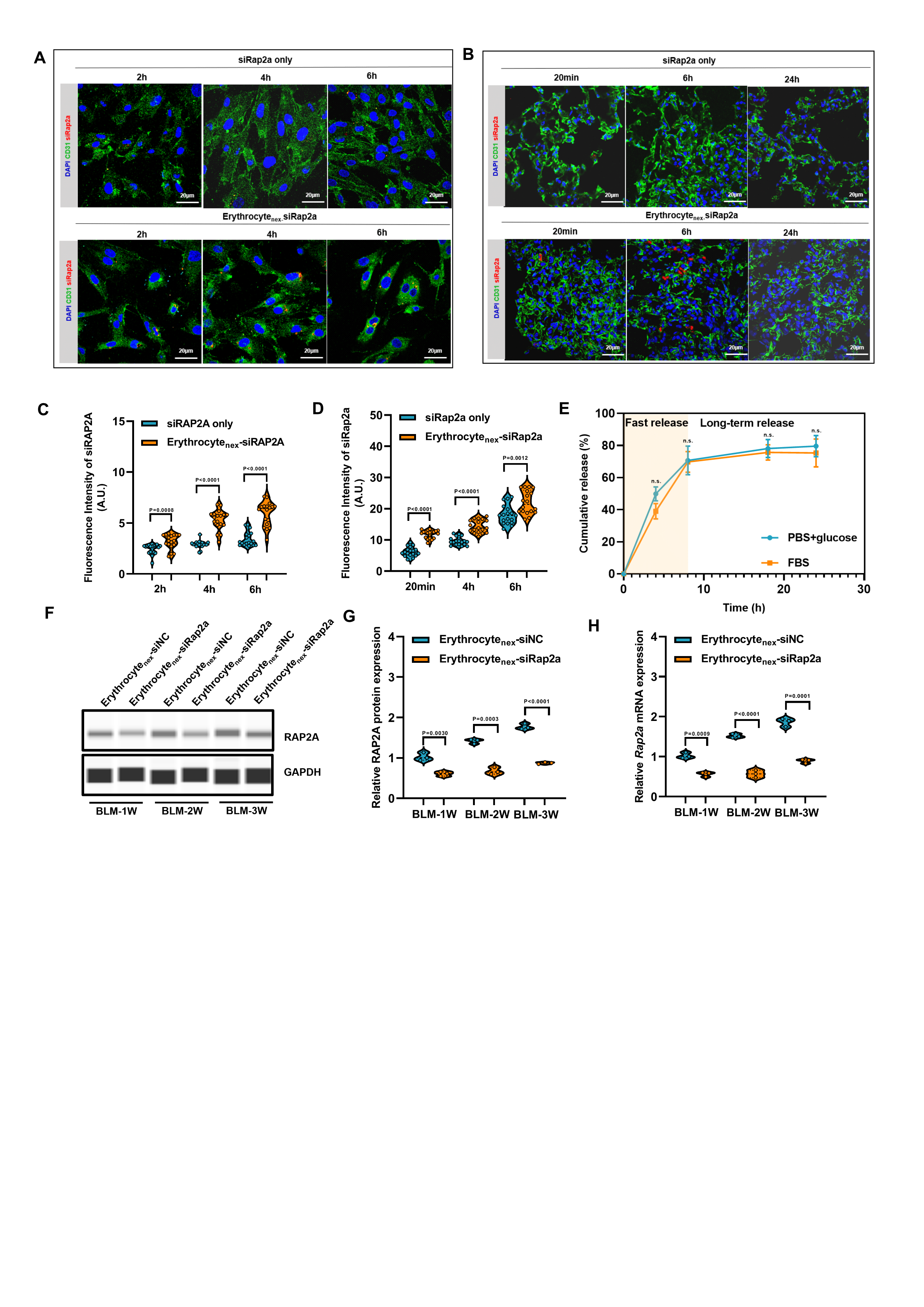


**Supplementary Figure S7. In vivo uptake, release kinetics, and gene silencing efficiency of erythrocyte_n_ₑₓ-siRap2a.** (A) Confocal microscopy of HUVECs incubated with erythrocyte_nex_-siRap2a or free siRNA for 2, 4, and 6 h, showing enhanced intracellular uptake. (B) Immunofluorescence staining of lung sections at 20 min, 6 h, and 24 h after intravenous injection demonstrating siRap2a uptake by pulmonary CD31⁺ endothelial cells. (C–D) Quantification of intracellular fluorescence intensity in vitro (C) and in vivo (D), indicating time-dependent accumulation of siRap2a. (E) Cumulative siRNA release profiles showing biphasic release kinetics, with an initial burst release in PBS supplemented with glucose and sustained release in fetal bovine serum (FBS). (F–H) RAP2A protein (F, G) and *Rap2a* mRNA (H) levels in lung tissues from BLM-treated mice following erythrocyte_nex_-siRap2a or control treatment at 1, 2, and 3 weeks, demonstrating effective in vivo gene silencing. Data are presented as mean ± SEM. Exact *P* values are indicated. **Table S1.** qRT-PCR primer sequences.

| Gene | | Primers | Sequences (5′–3′) |
| --- | --- | --- | --- |
| Human | homo-RAP2A | Forward | ATGCGCGAGTACAAAGTGGT |
|  |  | Reverse | GCGACGAATCCACCTCGAT |
|  | homo-MAP4K4 | Forward | GACTCCCCTGCAAAAAGTCTG |
|  |  | Reverse | GTCCATAGGTGCCATTTCCAA |
|  | homo-VCAM1 | Forward | GGGAAGATGGTCGTGATCCTT |
|  |  | Reverse | TCTGGGGTGGTCTCGATTTTA |
|  | homo-GAPDH | Forward | GGAGCGAGATCCCTCCAAAAT |
|  |  | Reverse | GGCTGTTGTCATACTTCTCATGG |
| Mouse | mus-Rap2a | Forward | AATACGACCCCACCATCGAG |
|  |  | Reverse | ACCTTCTCATACCGCTTCACG |
|  | mus-Gapdh | Forward | AGGTCGGTGTGAACGGATTTG |
|  |  | Reverse | GGGGTCGTTGATGGCAACA |
